# Pure Boric Acid Does Not Show Room‐Temperature Phosphorescence (RTP)

**DOI:** 10.1002/anie.202200599

**Published:** 2022-02-19

**Authors:** Zhu Wu, Juan Carlos Roldao, Florian Rauch, Alexandra Friedrich, Matthias Ferger, Frank Würthner, Johannes Gierschner, Todd B. Marder

**Affiliations:** ^1^ Institut für Anorganische Chemie and Institute for Sustainable Chemistry & Catalysis with Boron Julius-Maximilians-Universität Würzburg Am Hubland 97074 Würzburg Germany; ^2^ Madrid Institute for Advanced Studies IMDEA Nanoscience Calle Faraday 9, Campus Cantoblano 28049 Madrid Spain; ^3^ Institut für Organische Chemie and Center for Nanosystems Chemistry Julius-Maximilians-Universität Würzburg Am Hubland 97074 Würzburg Germany

**Keywords:** Boric Acid, Impurity, Phosphorescence, TD-DFT, Triplet

## Abstract

Boric acid (BA) has been used as a transparent glass matrix for optical materials for over 100 years. However, recently, apparent room‐temperature phosphorescence (RTP) from BA (crystalline and powder states) was reported (Zheng et al., *Angew. Chem. Int. Ed*. **2021**, *60*, 9500) when irradiated at 280 nm under ambient conditions. We suspected that RTP from their BA sample was induced by an unidentified impurity. Our experimental results show that pure BA synthesized from B(OMe)_3_ does not luminesce in the solid state when irradiated at 250–400 nm, while commercial BA indeed (faintly) luminesces. Our theoretical calculations show that neither individual BA molecules nor aggregates would absorb light at >175 nm, and we observe no absorption of solid pure BA experimentally at >200 nm. Therefore, it is not possible for pure BA to be excited at >250 nm even in the solid state. Thus, pure BA does not display RTP, whereas trace impurities can induce RTP.

## Graphical abstract

For over 100 years, boric acid (BA) has been known to serve as an excellent glass matrix for luminescent chromophores. However, in contrast to a recent report, pure BA does NOT display RTP when excited at 280 nm, and it is demonstrated experimentally that it does not absorb at *λ*>200 nm. Theoretical studies further reveal that pure BA cannot absorb at *λ*>175 nm. Thus, an impurity must be responsible for the apparent RTP in the previous report.

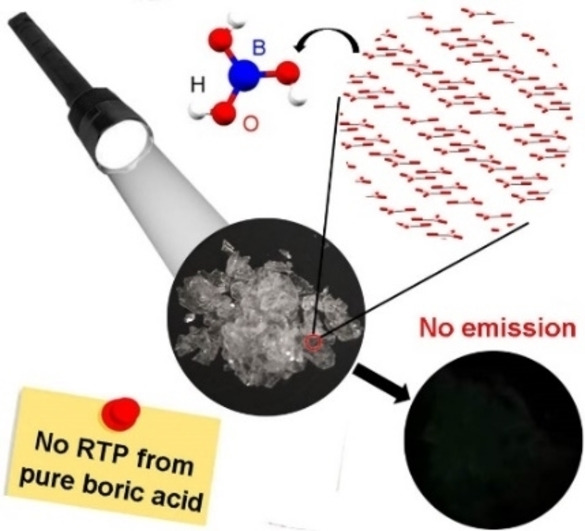

## Introduction

Boric acid (BA) made from borax was first reported in 1832.^[1]^ In the following 100 years, reactions of BA with other inorganic salts were continuously investigated.^[2]^ Since 1920, researchers have been using BA as a glass matrix for optical materials, and have reported that pure BA does not luminesce.^[3]^ Because of its high electron affinity, BA is a suitable matrix material for radical cations of aromatics such as naphthalene and anthracene to study their absorption spectra.^[4]^ With its high degree of rigidity to suppress vibration‐ and rotation‐assisted nonradiative decay,^[5]^ delayed fluorescence^[6]^ and phosphorescence^[7]^ of aromatic hydrocarbons doped into a BA glass matrix can be realized even at room temperature. Due to these advantages, BA glass has become one of the most convenient and attractive media for studying the photophysical behavior of aromatic hydrocarbons.^[8]^ In the last two decades, purely organic materials which display persistent room temperature phosphorescence (RTP) have received tremendous interest because of their low cost, easy fabrication, and high biological compatibility,^[9]^ which led to a wide variety of applications in biological imaging,^[10]^ organic light‐emitting diodes (OLEDs),^[11]^ and security printing technology.^[12]^ Unlike for metal complexes, with strong spin‐orbit coupling (SOC), intersystem crossing (ISC) in organic compounds, which plays an important role in phosphorescence emission, is typically less effective.^[13]^ In addition, under ambient conditions, nonradiative deactivation by ISC and consecutive thermal relaxation as well as quenching by species such as O_2_, rapidly deactivate the triplet excitons.^[14]^ Therefore, on the one hand, introducing heavy atoms to enhance the SOC and incorporating non‐metal heavy atoms or carbonyl groups with lone pairs^[14]^ are useful approaches to develop RTP systems with high performance.^[15]^ On the other hand, suppressing the nonradiative decay path through vibrational‐assisted relaxation and avoiding collisions with O_2_ by crystallization or polymerization are also key to realizing persistent afterglow.[^5^, ^16^] A variety of organic RTP materials were developed by utilizing the above strategies. However, concern for the existence of impurities and their role in contributing to the observed phosphorescence has been discussed since the 1920s.[^3b^, ^17^] Organic crystal impurities and their luminescence sensitization have been intensively analyzed in the following decades in solid state physics;^[17]^ however, this was largely overlooked in the materials science community, and has been raised again only recently.^[18]^ For example, Liu's group identified and isolated a carbazole isomer which is responsible for many carbazole‐related phosphors made from commercial carbazole sources^[19]^ and Ma and co‐workers designed a number of trace impurity‐induced bicomponent RTP systems based on derivatives.^[20]^ Zhang's group reported that a side product produced by reaction with the DMF solvent resulted in red RTP when doped into phthalimide hosts.^[21]^ All of the above results show that even trace impurities can dramatically influence the photophysical properties of organic systems.

**Figure 1:**
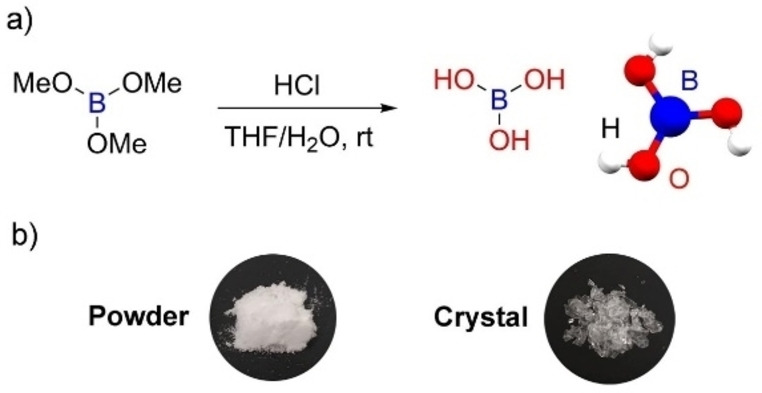
a) Synthesis of boric acid from B(OMe)_3_. b) Photographs of BA as a ground powder and as crystals, taken under daylight. The ground powder retains crystallinity.

In 2021, P. Wu and co‐workers reported that “pure” BA exhibits RTP with a lifetime of up to 0.6 s in the crystalline state when irradiated at 280 nm under ambient conditions.^[22]^ Based on our experience in research on 3‐coordinate boron‐containing optical materials,^[23]^ and that of other leading research groups,^[24]^ and increasing numbers of reports on so‐called “unconventional” luminescence phenomena,^[25]^ as well as the history noted above, we were suspicious of the reported phenomenon. Therefore, we synthesized pure BA from B(OMe)_3_ to examine its optical properties, including any possible RTP, as crystals and ground powder (Figure 1a), noting that the ground powder retains crystallinity, i.e., it is not amorphous. When exposed to 254 nm UV light, in contrast to the previous report^[22]^ there is no emission observed from either crystalline or powdered BA prepared as noted above. Upon turning off the UV light, the pure BA solid samples do not show any afterglow, indicating that the recently reported RTP from BA was induced by an unknown impurity in their sample. Importantly, and in contrast, a commercial sample of BA of the type employed in the previous study^[22]^ does show RTP in our spectrophotometers, as well as a very broad absorption starting at ca. 375 nm, which must be due to an impurity. In addition, our experimental and theoretical investigations suggest that there is no possibility of pure BA to show RTP at the excitation wavelength of 280 nm employed in reference [22], as the pure material has no absorption in that region of the spectrum (200–450 nm).

## Results and Discussion

**Figure 2:**
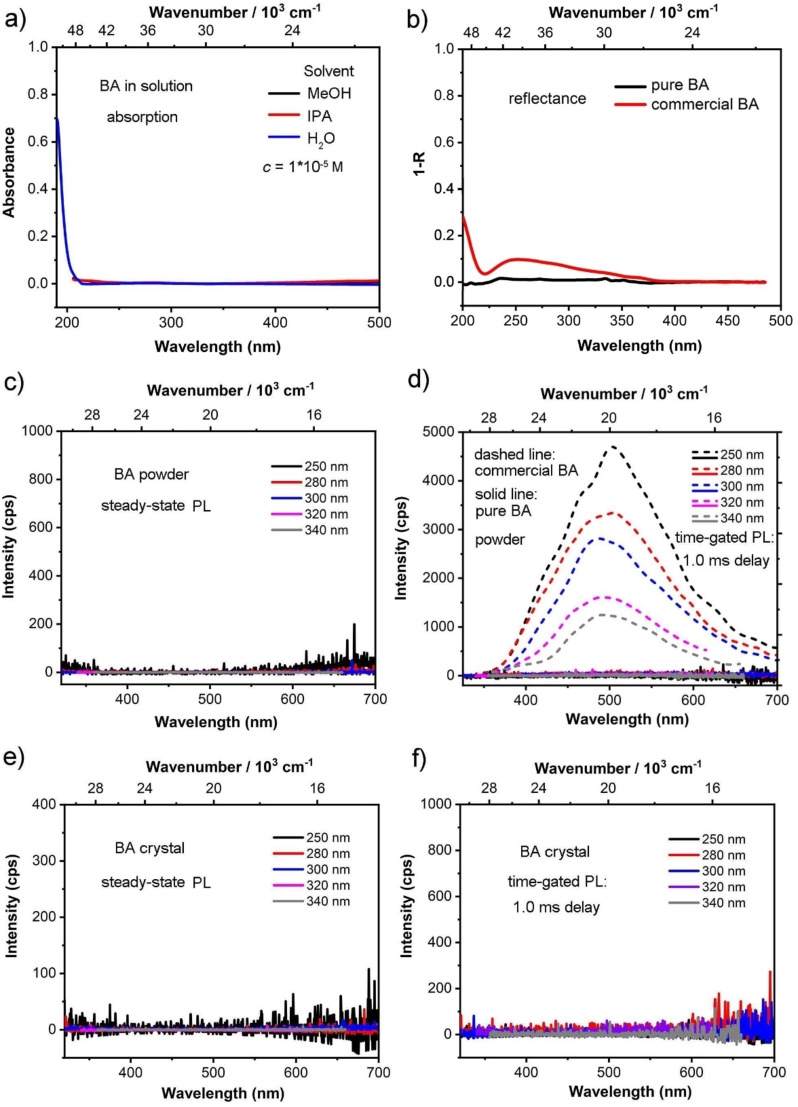
a) UV/Vis absorption spectra of pure BA in MeOH, isopropanol and H_2_O solutions (*c*=10^−5^ M). The onset of absorption at ca. 205 nm is due to the solvent cut‐off. b) UV/Vis absorption spectra (R=Reflectance) of solid samples of pure, crystalline BA (black) and commercial BA (red). Steady‐state photoluminescence (PL) (c) and time‐gated PL: 1.0 ms delay (d) spectra of commercial BA (dashed line) and pure BA (solid line) as a ground powder (not amorphous) at different excitation wavelengths under the same conditions. Photoluminescence (e) and time‐gated (1.0 ms delay) emission (f) spectra of pure, crystalline BA at different excitation wavelengths.

We synthesized and purified a sample of BA starting from B(OMe)_3_, and obtained clean ^11^B NMR spectra of our sample, both in solution and in the solid state (for details, see the Supporting Information). We then measured the absorption spectra of BA in methanol, isopropanol, and water solutions at concentrations of 10^−5^ M (Figure 2a). The cut‐off wavelengths of methanol and isopropanol are 205 nm. There was no absorption peak observed at wavelengths longer than 205 nm, which indicates that aromatic species are not present in our sample of BA. The cut‐off wavelength of water is 190 nm. There is also no absorption peak observed in the spectrum at >200 nm. Even upon increasing the concentration of BA in methanol to 10^−4^ or 10^−3^ M, no absorbance at >205 nm was observed (Figure S1). Solid state absorption spectra were then recorded on a Varian Cary 5 UV/Vis‐NIR spectrophotometer using a “praying mantis diffuse reflectance” assembly that allowed us to record absorption spectra to 200 nm. Absorption and excitation spectra of anthracene in the solid state were also recorded as a reference (Figures S2 and S3). What is clear is that the spectrum of pure BA does not show any absorption in the region between 200 and 485 nm. In contrast, commercial BA (Sigma–Aldrich: 99.999 % trace metals basis, the commercial material employed by Wu et al. with the highest purity as specified by the manufacturer) shows a broad absorption band with an onset at ca. 380 nm (Figure 2b), indicating the presence of impurities.

According to the study published by Wu et al.,^[22]^ upon changing the excitation wavelength from 280 to 365 nm, the phosphorescence intensity continuously decreased. Therefore, we measured the photoluminescent (PL) emission and time‐gated (1 ms) phosphorescence emission spectra of BA as both crystals and as a ground powder, again, noting that the powder retains crystallinity, with varied excitation wavelengths from 250 to 400 nm (Figures 2c–f and Figures S4–S9). However, as expected, neither the PL nor the time‐gated phosphorescence spectra of our pure BA show emission bands, either as a ground powder or as crystals. As a control experiment, we measured PL spectra of the commercial BA sample under the same experimental conditions; indeed, in this case a faint PL signal at room temperature was detected (see dashed lines in Figures 2d, and S11); the PL spectra are similar but somewhat redshifted^[26]^ and with a similar small blue shift upon going from room temperature to 77 K to that reported in Ref. [22]. It is further noted that the onset of PL (at ca. 370 nm) matches well with the absorption onset (at ca. 380 nm) of commercial BA (Figure 2b); this further indicates that the PL originates from the impurity which gives rise to these absorption features. Thus, our comparison of the absorption and PL features of pure and commercial BA undoubtedly proves that even trace impurity levels in commercial BA are sufficient to lead to RTP.

As in Ref. [22], we examined the commercial BA sample by both ^1^H NMR (using D_2_O, CD_3_OD, CD_2_Cl_2_, and THF‐d_8_ as solvents) and HRMS, but did not obtain evidence for impurity levels larger than the detection limit. Nonetheless, it is clear that the commercial BA sample must contain some form of impurity which gives rise to the absorption and emission observed both in Ref. [22] and by us. We stress, in this context, that even trace amounts of luminescent impurities are easily detected by PL spectroscopy due to the exceptional sensitivity of the method. The nature and amount of this impurity remain unknown; however, we point out again that our synthesis of pure BA is based on B(OMe)_3_, while industrial synthesis routes usually rely on reaction of boron‐containing minerals with sulfuric acid.^[27]^

**Figure 3:**
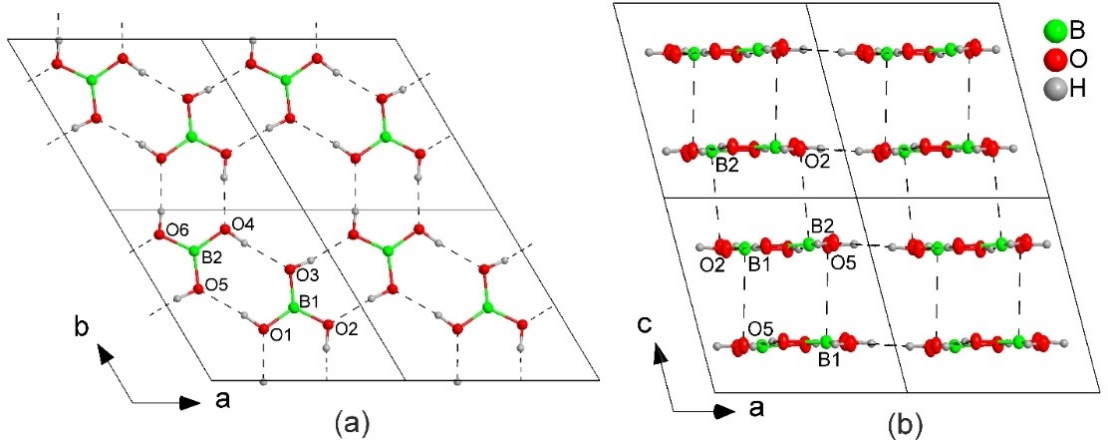
a) One sheet of hydrogen‐bonded BA molecules perpendicular to the *c* axis at 100 K. Four unit cells are shown within the (001) plane. b) Four unit cells (2×1×2) of the crystal structure of BA projected along the *b* axis at 100 K. The closest interplanar B⋅⋅⋅O contacts [Å] are illustrated by dashed lines: B1⋅⋅⋅O5 3.064(2), B2⋅⋅⋅O2 3.031(2).

Next, we obtained crystals of pure BA from methanol and collected single‐crystal X‐ray diffraction data both at 100 K and at room temperature (Table S2). The crystal structures obtained are consistent with the published crystal structure of BA (ICSD‐61354),^[28]^ which belongs to the triclinic crystal system (space group *P* $\bar{1}$ ). The BA molecules are arranged in layers which are perpendicular to the *c* axis. Within one layer, they interact via strong hydrogen bonds with H⋅⋅⋅O distances in the range of 1.858–1.873 Å and O−H⋅⋅⋅O angles of 172.2–174.8° at 100 K, forming six‐membered rings (Figures 3a and S18). The layers weakly interact via relatively short interplanar B⋅⋅⋅O contacts (3.031(2) and 3.064(2) Å at 100 K, Figure 3b). Unit‐cell parameters determined at 100 K and room temperature are similar. The direction of the largest thermal expansion upon temperature increase is along the *c* axis, which is the stacking direction of the weakly interacting layers (B⋅⋅⋅O=3.153(3) and 3.190(3) Å at 296 K). This results in an overall unit‐cell volume expansion from 262.97(3) Å^3^ at 100 K to 273.50(19) Å^3^ at room temperature upon temperature increase.

It needs to be noted here that the authors in Ref. [22] did not determine the crystal structure of their BA sample, but used the structural model of a trigonal boric acid polymorph, which can be found in the CCDC database (CCDC‐1500066) and refers to the report from Kurakevych and Solozhenko,^[29]^ for discussion in their paper. This is the only crystal structure of BA deposited in the CCDC database, while all other crystal structures of BA are deposited in the ICSD database (e.g., ICSD‐61354 for the triclinic polymorph). However, Kurakevych and Solozhenko^[29]^ used the structural model of Shuvalov and Burns^[30]^ for a rare polymorph of BA, H_3_BO_3_‐3*T*, in order to interpret their multiphase powder X‐ray diffraction pattern obtained from a high‐pressure, high‐temperature synthesis of a boron subnitride. The H_3_BO_3_‐3*T* polymorph has trigonal symmetry (space group *P*3_2_, ICSD‐281322) and was characterized by Shuvalov and Burns^[30]^ as an unexpected product crystallizing in addition to the triclinic H_3_BO_3_‐2*A* polymorph in an attempt to synthesize new sodium uranyl borate compounds. The authors in Ref. [22] mentioned a good match of the powder X‐ray diffraction pattern obtained from their boric acid sample with the structural model of trigonal H_3_BO_3_‐3*T* (see Figure S5 in Wu et al.^[22]^). However, the trigonal model does not describe all observed reflections of the diffraction pattern. Instead, the diffraction pattern can be nicely interpreted as the crystal structure of triclinic H_3_BO_3_‐2*A*, as is obvious from a simulation of the powder X‐ray diffraction patterns of both polymorphs (Figure S20). Hence, we conclude that the BA sample in Ref. [22] was indeed the same triclinic polymorph of BA as that in our own study.

Further negative evidence for an intrinsic cause of the appearance of RTP in solid BA arises from quantum‐chemical calculations. This was in fact done in Ref. [22], based on time‐dependent density functional theory (TD‐DFT); however, the results were interpreted in an erroneous way. In fact, the authors focused on the natural transition orbital (NTO) topologies, which indicated some through‐space interactions for BA clusters in the (110) plane. Furthermore, they showed how the TD‐DFT calculated absorption spectra experience a bathochromic shift upon clustering. Nevertheless, the authors failed to discuss the fact that their calculated maximum absorption wavelength of the BA clusters still resides in the far UV (reaching just 140 nm); under no circumstances can this be the source of their observed RTP in the visible range.^[31]^

**Figure 4:**
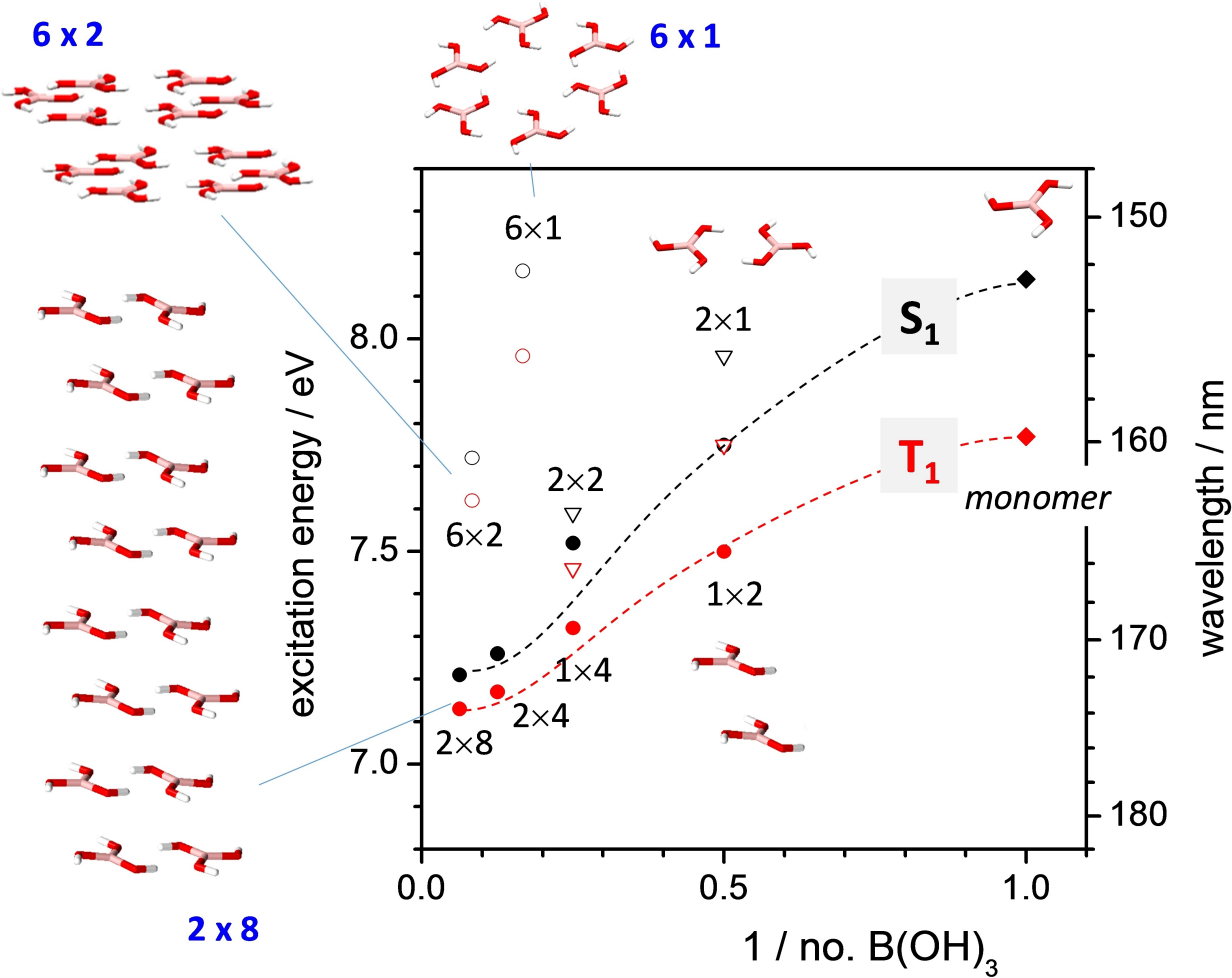
Vertical transition energies for the lowest excited singlet and triplet states (S_1_, T_1_) of BA and clusters thereof, as calculated by TD‐DFT; dashed lines are guides for the eyes.

In any case, as the calculated cluster sizes were not very large in the previous calculations, and one may argue that the results could depend on the functional employed, we calculated herein larger clusters, and extended them along the crystallographic *c* axis (see Figure 4; for DFT calculated natural transition orbitals (NTOs), see Figure S15). In this case, the B3LYP functional was employed, which tends to underestimate electronic transition energies,^[32]^ and thus may define a lower limit for the appearance of BA cluster absorption. As shown in Figure 4, clustering of boric acid units in fact generally leads to a bathochromic shift of the electronic S_1_ and T_1_ transition energies; the shift becomes particular large if the clusters are extended in the *c*‐direction. However, a rapid saturation is observed, so that the maximum shift of the clusters vs. the monomer amounts to ca. 0.65 eV for T_1_ and 0.95 eV for S_1_. Although the shift is considerable, we emphasize that the absorption remains in the deep UV (above 7 eV; 177 nm), in good agreement with our experimental results.

Our study has clearly shown that carefully purified BA, synthesized from B(OMe)_3_, does not show any intrinsic phosphorescence in the solid state, in particular, in the visible range; TD‐DFT calculations fully support our experimental results. In contrast, commercial BA evidently contains traces of (unknown) luminescent impurities, giving rise to blue‐colored RTP. Impurity sources could well be side products from synthesis, including impurities in any solvents used, sample contamination, or photoproducts. Such (luminescent) trap states in molecular solids^[33]^ can be directly excited if the concentration and absorption coefficient of the impurities are sufficiently high, which is in fact the case for commercial BA, as demonstrated in the current work (see Figure 2b).

Nevertheless, we emphasize that trap luminescence in molecular solids can also be observed if the trap absorption is very low (i.e., below the detection limit) but absorption of the host (i.e. the bulk material) is sufficiently high, as the traps may then very efficiently be reached by exciton migration,[^34^, ^35^, ^36^] down to molar ratios as low as 10^−8^.[^5^, ^37^, ^38^] We emphasize that a whole generation of solid state physicists was engaged to clarify PL phenomena in organic solids and to disentangle pristine PL of a specific material from dopant/impurity‐related PL.[^17b^, ^39^] This has been achieved for materials, e.g., naphthalene and perylene, by sophisticated purification techniques such as zone refinement by the Bridgman method,^[40]^ but obviously has remained unnoticed by many in the currently active community of chemists working on related phenomena. If the host is non‐luminescent, exclusive sensitized PL is observed; otherwise dual PL will be perceived.^[33c]^ These sensitizer concentrations can be so low that an unambiguous proof for the absence of trap states by standard analytical methods is not possible; instead, only a negative proof can be given. This type of “falsification procedure” (negative proof) was successfully followed in the current work; such proof is tedious, but of tremendous importance as it gives a clear, general warning for reports on uncommon luminescence phenomena. In fact, as stated earlier,^[5]^ it seems likely that many reports on so‐called “unconventional luminescence” from solid‐state samples of non‐conjugated chromophores,^[25]^ may suffer from impurity problems as reported here for commercial BA.

## Conclusions

Boric acid (BA) can be used as an excellent glass matrix, which, when pure, does not display any fluorescence or phosphorescence in the solid state. Our absorption spectra, measured both in solution and in the solid state, clearly show that it is not possible for BA to be excited at wavelengths longer than 200 nm. Our theoretical studies further suggest that the excitation of BA cannot occur at wavelengths longer than 175 nm. Our emission measurements also prove that there is no luminescence from pure BA in the visible region of the spectrum. All above results prove that the reported RTP from commercial BA must have been induced by unknown impurities. We are indeed of the opinion that such “falsification procedures” to explore all possible avenues for “negative proof” should be attempted in all original research, especially on systems which display unusual luminescence properties, for example, and for which there is no reason to expect such properties, as this represents good scientific practice. In addition, as we show by discussion of the ca. 100‐year history of the use of BA glasses as hosts for RTP systems, it is incumbent upon researchers to search and read the literature carefully, and to cite an appropriate list of relevant publications.

## Conflict of interest

The authors declare no conflict of interest.

1

## Supporting information

As a service to our authors and readers, this journal provides supporting information supplied by the authors. Such materials are peer reviewed and may be re‐organized for online delivery, but are not copy‐edited or typeset. Technical support issues arising from supporting information (other than missing files) should be addressed to the authors.

Supporting Information

Supporting Information

Supporting Information

Supporting Information

Supporting Information

## Data Availability

The data that support the findings of this study are available in the Supporting Information of this article.
