# Supplementary material for: Pure Boric Acid Does Not Show Room‐Temperature Phosphorescence (RTP)
Source: Angew Chem Int Ed Engl. 2022 Feb 19;61(15):e202200599. doi: 10.1002/anie.202200599 (PMC9305524; doi:10.1002/anie.202200599)

checkCIF/PLATON report

Structure factors have been supplied for datablock(s) ZhWu072_AF_a

THIS REPORT IS FOR GUIDANCE ONLY. IF USED AS PART OF A REVIEW PROCEDURE FOR PUBLICATION, IT SHOULD NOT REPLACE THE EXPERTISE OF AN EXPERIENCED CRYSTALLOGRAPHIC REFEREE.

No syntax errors found.   CIF dictionary   Interpreting this report

Datablock: ZhWu072_AF_a

Bond precision: O- B = 0.0037 A   Wavelength=0.71073

Cell:	a=7.032(2)	b=7.045(4)	c=6.5737(19)
	alpha=92.51(3)	beta=101.19(2)	gamma=119.814(17)
Temperature:	296 K		

	Calculated	Reported
Volume	273.4(2)	273.4(2)
Space group	P -1	P -1
Hall group	-P 1	-P 1
Moiety formula	B H3 O3	B H3 O3
Sum formula	B H3 O3	B H3 O3
Mr	61.83	61.83
Dx,g cm-3	1.502	1.502
Z	4	4
Mu (mm-1)	0.155	0.155
F000	128.0	128.0
F000'	128.12	
h,k,lmax	8,8,8	8,8,8
Nref	1119	1100
Tmin,Tmax	0.944,0.954	0.666,0.745
Tmin'	0.938	

Correction method= # Reported T Limits: Tmin=0.666 Tmax=0.745
AbsCorr = MULTI-SCAN

Data completeness= 0.983   Theta(max)= 26.368

R(reflections)= 0.0471(1016)   wR2(reflections)= 0.1577(1100)

S = 1.247   Npar= 79

The following ALERTS were generated. Each ALERT has the format
test-name_ALERT_alert-type_alert-level.
Click on the hyperlinks for more details of the test.

Alert level C

```
PLAT155_ALERT_4_C The Triclinic Unitcell is NOT Reduced ..........     Please Do !
PLAT906_ALERT_3_C Large K Value in the Analysis of Variance ......      6.500 Check
PLAT911_ALERT_3_C Missing FCF Refl Between Thmin & STh/L=    0.600         19 Report
```

Alert level G

```
PLAT007_ALERT_5_G Number of Unrefined Donor-H Atoms .............          6 Report
PLAT913_ALERT_3_G Missing # of Very Strong Reflections in FCF ....          2 Note
PLAT933_ALERT_2_G Number of OMIT Records in Embedded .res File ...          3 Note
PLAT941_ALERT_3_G Average HKL Measurement Multiplicity ...........        3.9 Low
```

0 **ALERT level A** = Most likely a serious problem - resolve or explain
0 **ALERT level B** = A potentially serious problem, consider carefully
3 **ALERT level C** = Check. Ensure it is not caused by an omission or oversight
4 **ALERT level G** = General information/check it is not something unexpected

0 ALERT type 1 CIF construction/syntax error, inconsistent or missing data
1 ALERT type 2 Indicator that the structure model may be wrong or deficient
4 ALERT type 3 Indicator that the structure quality may be low
1 ALERT type 4 Improvement, methodology, query or suggestion
1 ALERT type 5 Informative message, check

It is advisable to attempt to resolve as many as possible of the alerts in all categories. Often the minor alerts point to easily fixed oversights, errors and omissions in your CIF or refinement strategy, so attention to these fine details can be worthwhile. In order to resolve some of the more serious problems it may be necessary to carry out additional measurements or structure refinements. However, the purpose of your study may justify the reported deviations and the more serious of these should normally be commented upon in the discussion or experimental section of a paper or in the "special_details" fields of the CIF. checkCIF was carefully designed to identify outliers and unusual parameters, but every test has its limitations and alerts that are not important in a particular case may appear. Conversely, the absence of alerts does not guarantee there are no aspects of the results needing attention. It is up to the individual to critically assess their own results and, if necessary, seek expert advice.

Publication of your CIF in IUCr journals

A basic structural check has been run on your CIF. These basic checks will be run on all CIFs submitted for publication in IUCr journals (*Acta Crystallographica*, *Journal of Applied Crystallography*, *Journal of Synchrotron Radiation*); however, if you intend to submit to *Acta Crystallographica Section C* or *E* or *IUCrData*, you should make sure that full publication checks are run on the final version of your CIF prior to submission.

Publication of your CIF in other journals

Please refer to the *Notes for Authors* of the relevant journal for any special instructions relating to CIF submission.

PLATON version of 13/07/2021; check.def file version of 13/07/2021

Datablock ZhWu072_AF_a - ellipsoid plot

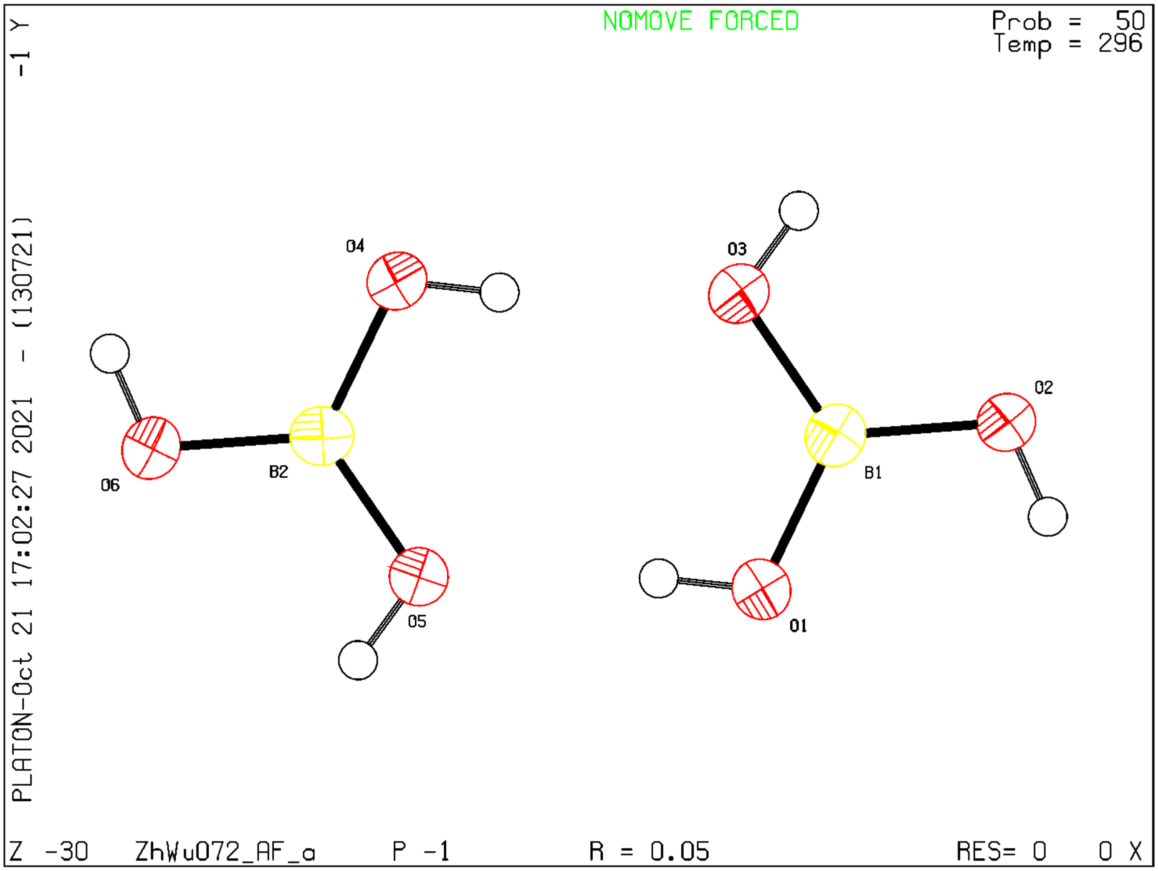

Supplement: Supplementary file 3 — Supporting Information [file ANIE-61-0-s002.pdf]
