# Supplementary material for: Pure Boric Acid Does Not Show Room‐Temperature Phosphorescence (RTP)
Source: Angew Chem Int Ed Engl. 2022 Feb 19;61(15):e202200599. doi: 10.1002/anie.202200599 (PMC9305524; doi:10.1002/anie.202200599)
Supplement: Supplementary file 5 — Supporting Information [file ANIE-61-0-s003.pdf]

Angewandte Chemie
Eine Zeitschrift der Gesellschaft Deutscher Chemiker

Supporting Information

Pure Boric Acid Does Not Show Room-Temperature Phosphorescence (RTP)

Z. Wu, J. C. Roldao, F. Rauch, A. Friedrich, M. Ferger, F. Würthner, J. Gierschner, T. B. Marder**

Table of Contents

I. General information....................S2

II. Experimental procedures and characterization....................S3

III. Photophysical characterization....................S5

IV. Results of the quantum chemical computations....................S14

V. Single-crystal X-ray diffraction....................S17

VI. ^{11}B NMR spectra....................S20

VII. References....................S23

VIII. Certificate of Analysis for the commercial sample of BA…………………….....S24

I. General information

The starting material, trimethyl borate, was purchased from Alfa Aesar and was used without further purification. Organic solvents for synthetic reactions were HPLC grade, further treated to remove trace water using an Innovative Technology Inc. Pure-Solv Solvent Purification System and deoxygenated using the freeze-pump-thaw method. The synthetic reaction was performed in air at room temperature. The solution ^{11}B NMR spectrum was measured on a Bruker Avance III HD 300 MHz (^{11}B, 96 MHz) NMR spectrometer. The ^{11}B{^{1}H} RSHE/MAS (Rotor Synchronized Hahn Echo/Magic Angle Spinning) solid-state NMR spectra were recorded on a Bruker Avance Neo WB 400 MHz spectrometer operating at 128.38 MHz for ^{11}B and 400.13 MHz for ^{1}H, using a 4 mm (o.d.) ZrO_2 rotor spinning at 14.8 KHz. The spectra were obtained using a new Bruker 4 mm CP/MAS ATM BBO/^{1}H&^{19}F iProbe. The selective 90° ^{11}B low power pulse was calibrated to 25 μs, to irradiate only the central transition. The acquisition time was 25 ms and the relaxation delay was set to 20 s. Chemical shifts were calibrated externally to the low field shift of adamantane (38.48 ppm). The ^{11}B solid-state spectra were simulated using the software package SOLA.[1]

General photophysical measurements. All measurements were performed in standard quartz cuvettes (1 cm × 1 cm cross-section). UV-visible absorption spectra were recorded using a Perkin Elmer LAMBDA 465 UV/Vis spectrophotometer. Solid state UV-vis absorption (reflectance) spectra were recorded using a Praying Mantis Diffuse Reflectance Accessory on a Varian Cary 5 UV-vis-NIR spectrophotometer with deuterium (UV) and quartz-iodide (vis) lamps as light sources, and PMT (UV-vis) and lead sulfide photocell (NIR) detectors. $BaSO_4$ was used as the reference. To ensure the reliability of the measurement of the boric acid sample, we recorded the reflectance and excitation spectra of anthracene, which shows excellent agreement between the two methods (Figures S2 and S3). Note the inverted relationship of the vibronic fine structure between the reflectance and excitation spectra of anthracene, a result of 'inner-filter' effects, which can become dramatic for solid samples due to their extremely high concentrations. See footnote 200 in reference 2. The emission spectra were recorded using an Edinburgh Instruments FLSP920 spectrometer equipped with a double monochromator, operating in right angle geometry mode. The time-gated (delay time 1.0 ms) phosphorescence spectra were measured using a μF920 pulsed 60 W Xenon microsecond flashlamp, with a repetition rate of 100 Hz at room temperature. All spectra were fully corrected for the spectral response of the instrument. For comparison, additional spectra were recorded on an Edinburgh Instruments FLSP980 spectrometer, and are labelled as such below.

Crystal structure determinations Crystals suitable for single-crystal X-ray diffraction were selected, coated in perfluoropolyether oil, and mounted on polyimide microloops. Diffraction data of boric acid at 100 K were collected on a Rigaku XTALAB Synergy S diffractometer with a semiconductor HPA-detector (HyPix-6000) using Cu-Kα radiation monochromated by multi-layer focusing mirrors. The crystal was cooled using an Oxford Cryostreams 800 low-temperature device. Diffraction data at ambient temperature (296 K) were collected on a BRUKER X8-APEX II diffractometer with a CCD area detector and multi-layer mirror monochromated Mo-Kα radiation. The images were processed and corrected for Lorentz-polarization effects and absorption as implemented in the Rigaku and Bruker software packages, respectively. The crystal structure was solved using the intrinsic phasing method (SHELXT)[3] and Fourier expansion technique. All non-hydrogen atoms were refined in anisotropic approximation, with hydrogen atoms 'riding' in idealized positions, by full-matrix least squares against F^2 of all data, using SHELXL[4] software and the SHELXLE graphical user interface.[5] The crystal structure was transformed to the non-standard setting reported for orthoboric acid by Gajhede *et al*.[6] Diamond[7] software was used for graphical representation. Other structural information was extracted using Mercury[8] and OLEX2[9] software. Crystal data and experimental details are listed in Table S2; full structural information has been deposited with Cambridge Crystallographic Data Centre. CCDC-2122545 ($B(OH)_3$ – 100 K) and 2122546 ($B(OH)_3$ – 296 K).

Computational Treatment. The ground state (S_0) geometry of boric acid was optimized by density functional theory (DFT) in vacuum, constrained to C_{3h} symmetry. Vertical transition energies for singlet and triplet states (S_n, T_n) were obtained by single point calculations on the S_0 geometry using time-dependent (TD) DFT. Implicit solvent effects were considered via the polarizable continuum model (PCM) using water as a solvent. Adiabatic energies were obtained by excited state geometry optimization of the S_1 state. For the cluster calculations, molecular structures from the X-ray crystallographic analysis were replaced by DFT-optimized ones, while intermolecular separations were retained from the experimental data. To obtain fully optimized S_0 and S_1 geometries in the crystal, quantum Mechanics/Molecular Mechanics (QM/MM) calculations were performed, using the ONIOM approach.[10] For this, the MM atoms were frozen in their experimental crystal structure positions and treated with the Dreiding force field. The atoms in the QM region were treated at the DFT level. Optimized geometries and their normal coordinates were only obtained for the QM region; for details, see Refs. [11, 12]. For all calculations, the B3LYP functional and 6-311g(d) basis set were used as defined in the Gaussian 09 program package.[13] CASSCF (complete active space self-consistent field) optimization of BA in the S_0 and S_1 states were performed using the OpenMolcas package[14,15] including all ππ* orbitals[16] and applying the ANO-RCC-VTZP basis-set.[17]

II. Experimental procedures and characterization

Synthesis of $B(OH)_3$

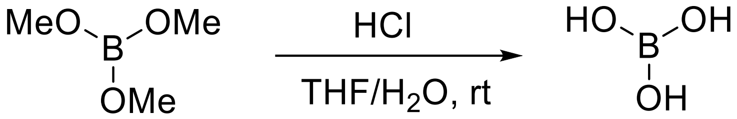

Trimethyl borate (0.9 mL, 8.0 mmol) was stirred in 65 mL of a 4:1 mixture of THF and water for 30 min, at which time aqueous hydrochloric acid (1N, 5.6 mL, 5.6 mmol) was added to the suspension. The reaction mixture was stirred at ambient temperature overnight. The reaction mixture was diluted with water (30 mL) and extracted with ethyl acetate (3 x 60 mL). The combined extracts were washed with water (2 x 30 mL) and brine (30 mL), dried over sodium sulfate, filtered, and concentrated to dryness by rotary evaporation. The residue was washed with small portions of hexane, to give a colorless, solid product. The solid $B(OH)_3$ was dissolved in methanol at room temperature. Then the solution was transferred to a refrigerator and recrystallized at least 3 times at -25 °C until pure material was obtained as indicated by ^{11}B NMR spectroscopy. Importantly, even the crude material prior to recrystallization did not show RTP.

Solution ^{11}B NMR (96 Hz, 298 K, D_2O): δ = 19.49 ppm.

Simulation parameters of the ^{11}B RSHE/MAS of lab-made $B(OH)_3$ (Figure S14): δ_{iso} = 20.1 ± 0.1 ppm (isotropic chemical shift), C_Q = 2506 ± 10 kHz (quadrupole coupling constant), ηQuad = 0.04 ± 0.02 (quadrupolar asymmetry parameter), LB = 80 Hz (line broadening parameter).

Simulation parameters of the ^{11}B RSHE/MAS of commercial $B(OH)_3$ (Figure S15): δ_{iso} = 20.2 ± 0.1 ppm (isotropic chemical shift), C_Q = 2510 ± 10 kHz (quadrupole coupling constant), ηQuad = 0.03 ± 0.02 (quadrupolar asymmetry parameter), LB = 110 Hz (line broadening parameter).

Single crystal growth: $B(OH)_3$ (62 mg, 1 mmol) was dissolved in 10 mL of MeOH in a test tube which was placed in a fumehood at room temperature in air, and the solvent was allowed to evaporate slowly. Colorless crystals suitable for single-crystal X-ray diffraction formed after *ca*. 24 h.

III. Photophysical characterization

All emission and excitation spectra were recorded on an Edinburgh Instruments FLS920 spectrophotometer unless otherwise indicated. Comparison spectra recorded on an Edinburgh Instruments FLS980 spectrophotometer are labelled as such.

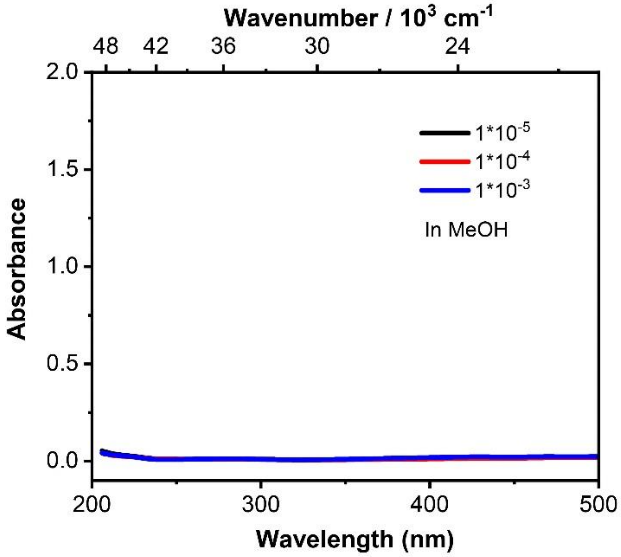

Figure S1. Absorption spectra of pure $B(OH)_3$ in MeOH solutions with varied concentrations (c = 10^{-5}, 10^{-4} and 10^{-3} M).

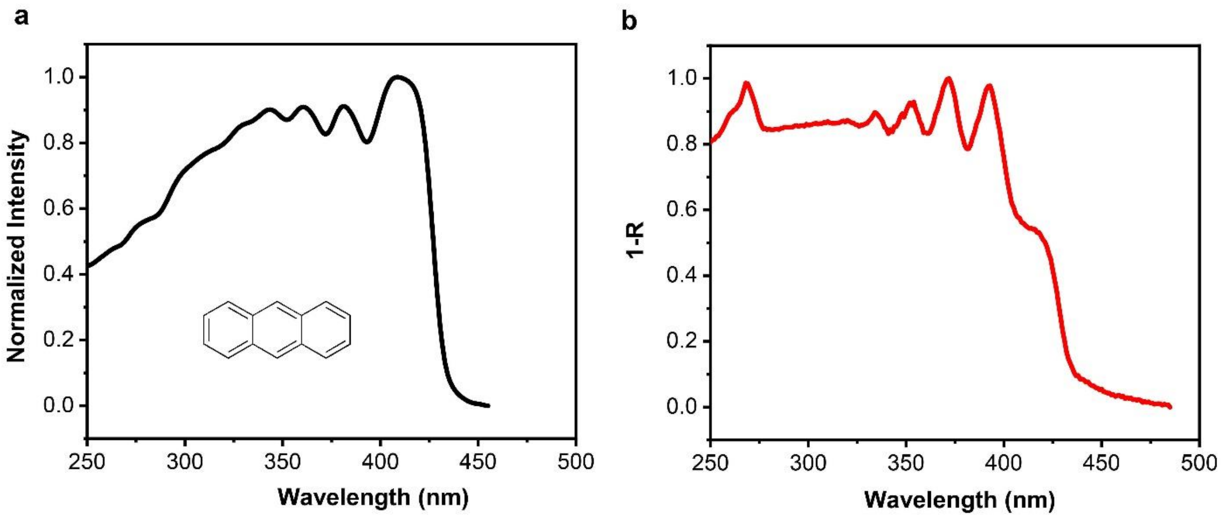

Figure S2. Excitation (a) and absorption (b) spectra of anthracene in the crystalline state at room temperature in air (R = Reflectance) as a reference.

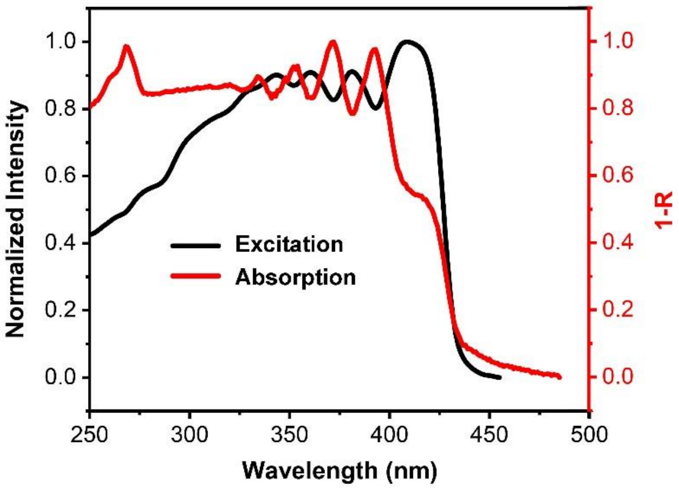

Figure S3. Comparison of excitation and absorption spectra of anthracene in the crystalline state at room temperature in air (R = Reflectance) as a reference.

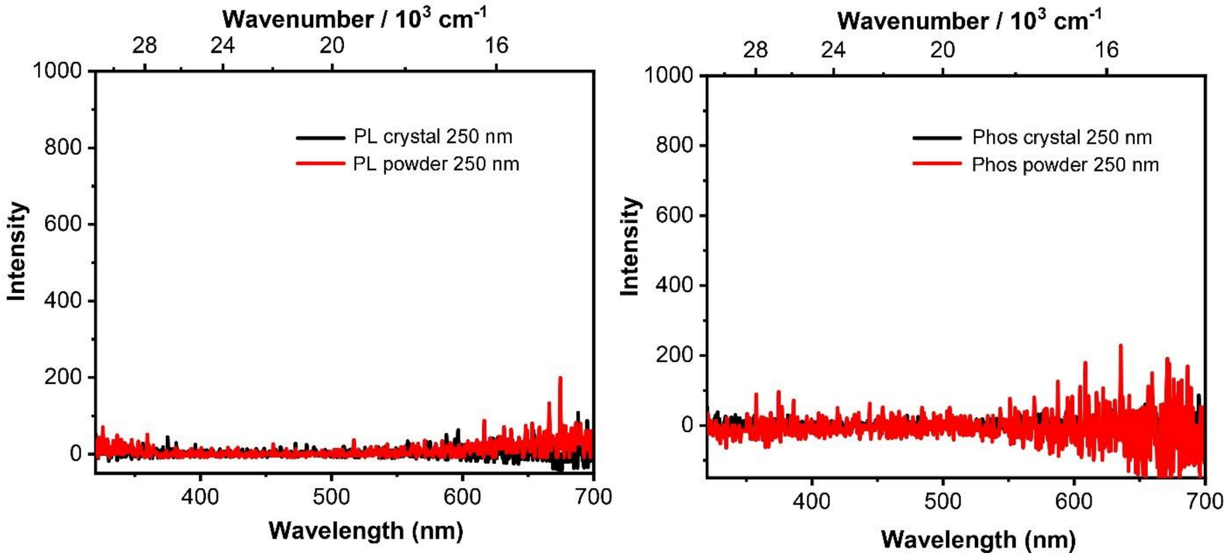

Figure S4. Photoluminescent emission (left) and time-gated (delay time: 1 ms) phosphorescence emission (right) spectra of pure $B(OH)_3$ in the crystal and powder states excited at 250 nm.

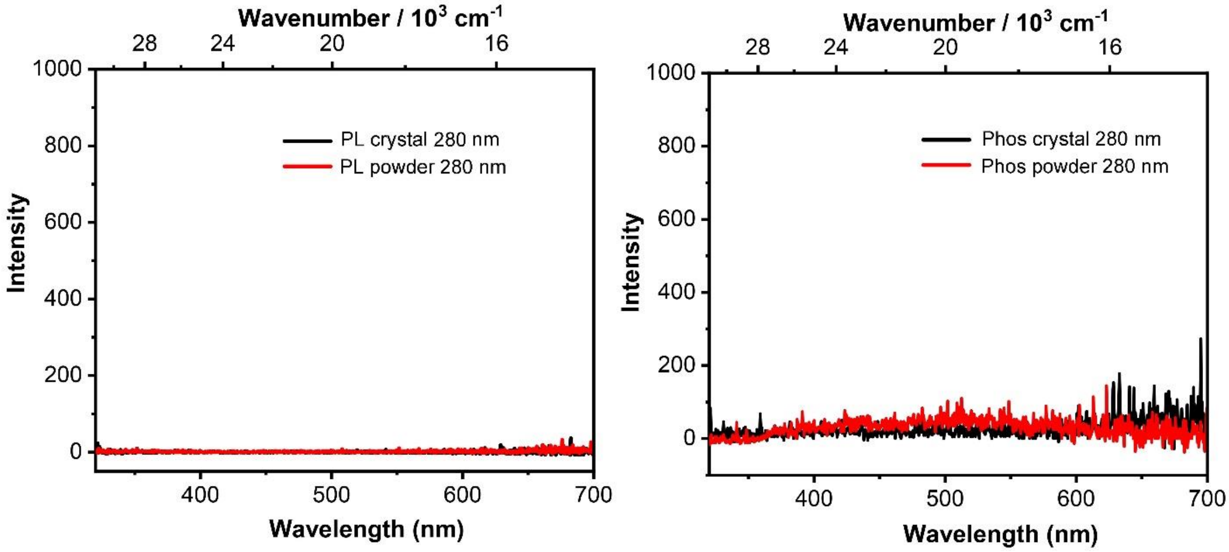

Figure S5. Photoluminescent emission (left) and time-gated (delay time: 1 ms) phosphorescence emission (right) spectra of pure $B(OH)_3$ in the crystal and powder states excited at 280 nm.

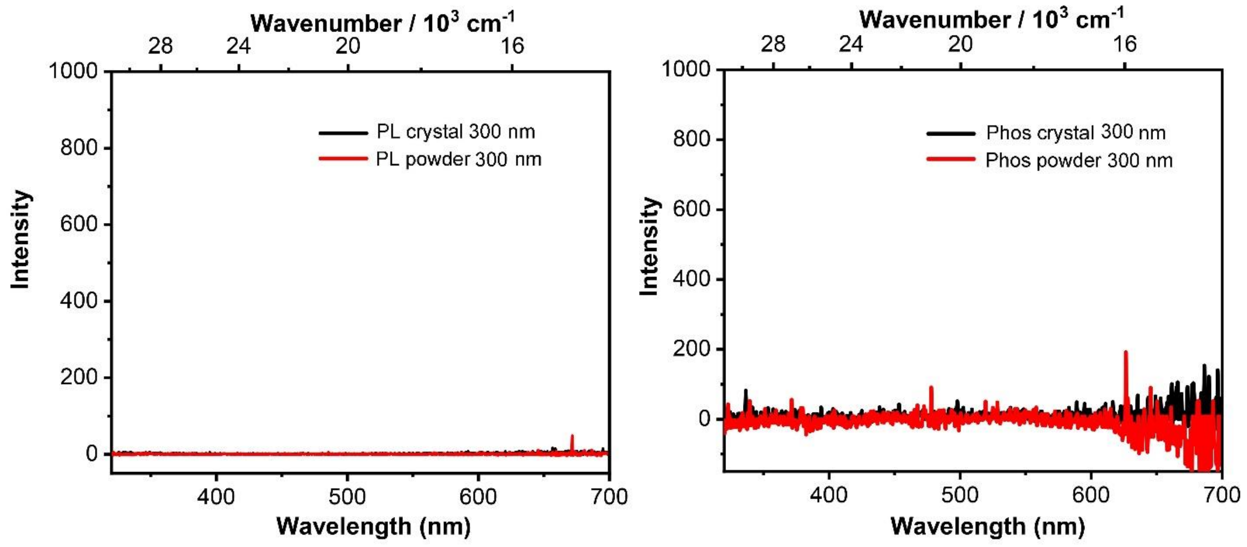

Figure S6. Photoluminescent emission (left) and time-gated (delay time: 1 ms) phosphorescence emission (right) spectra of pure $B(OH)_3$ in the crystal and powder states excited at 300 nm.

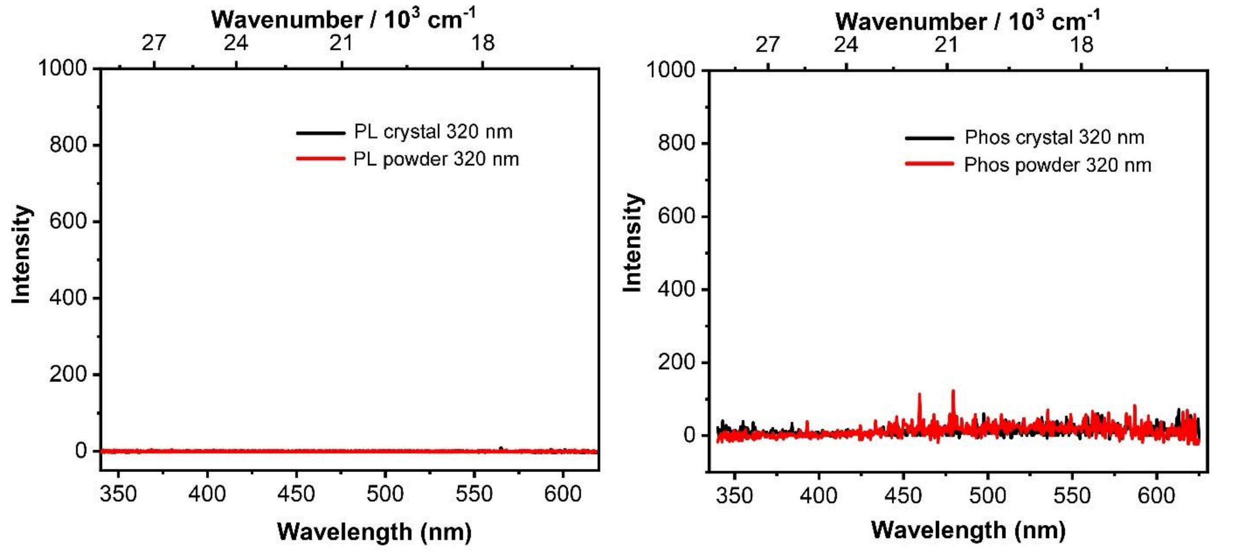

Figure S7. Photoluminescent emission (left) and time-gated (delay time: 1 ms) phosphorescence emission (right) spectra of pure $B(OH)_3$ in the crystal and powder states excited at 320 nm.

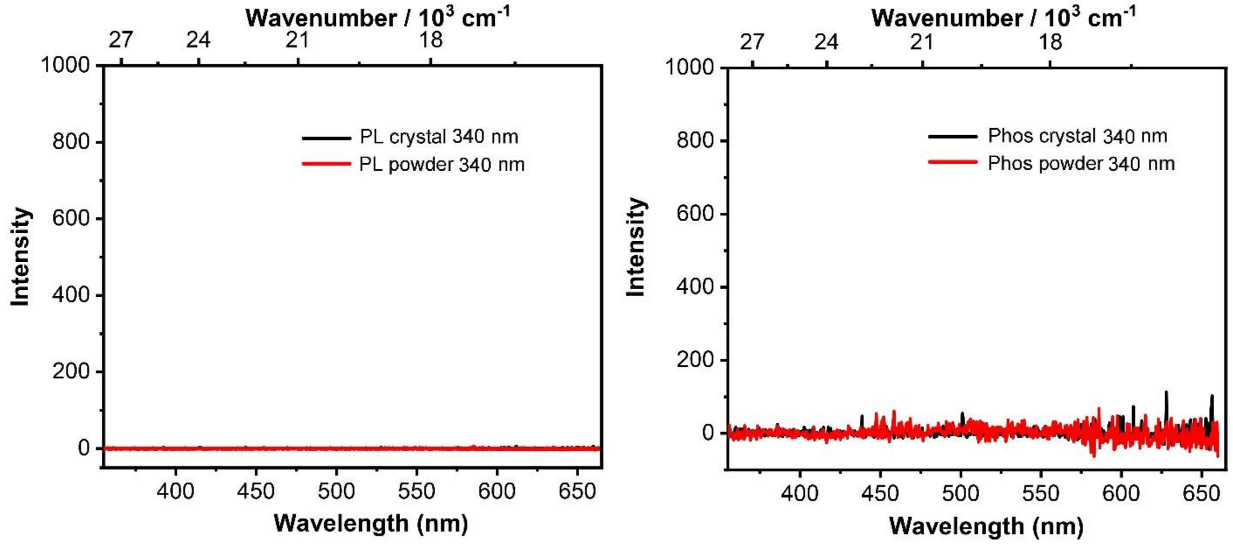

Figure S8. Photoluminescent emission (left) and time-gated (delay time: 1 ms) phosphorescence emission (right) spectra of pure $B(OH)_3$ in the crystal and powder state excited at 340 nm.

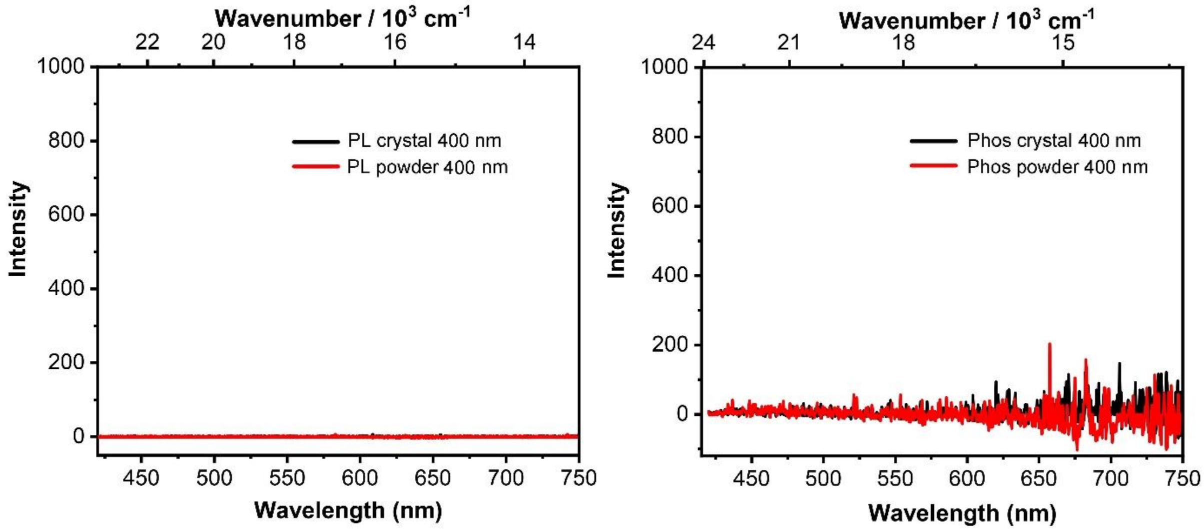

Figure S9. Photoluminescent emission (left) and time-gated (delay time: 1 ms) phosphorescence emission (right) spectra of pure $B(OH)_3$ in the crystal and powder states excited at 400 nm.

Additional photophysical data on pure BA and commercial BA (Sigma-Aldrich: 99.999%)

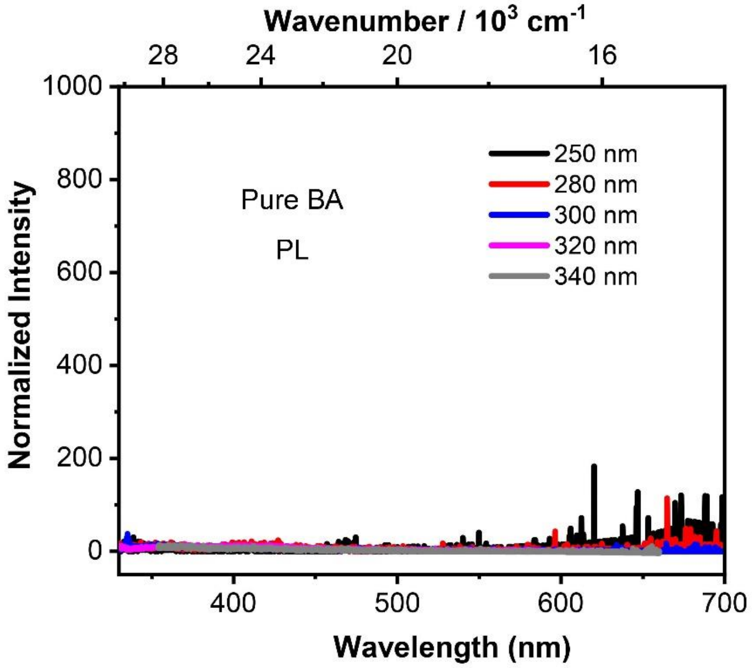

Figure S10. Normalized photoluminescence spectra of pure BA in crystalline state at different excitation wavelengths at room temperature on a different spectrophotometer (Edinburgh Instruments FLS980).

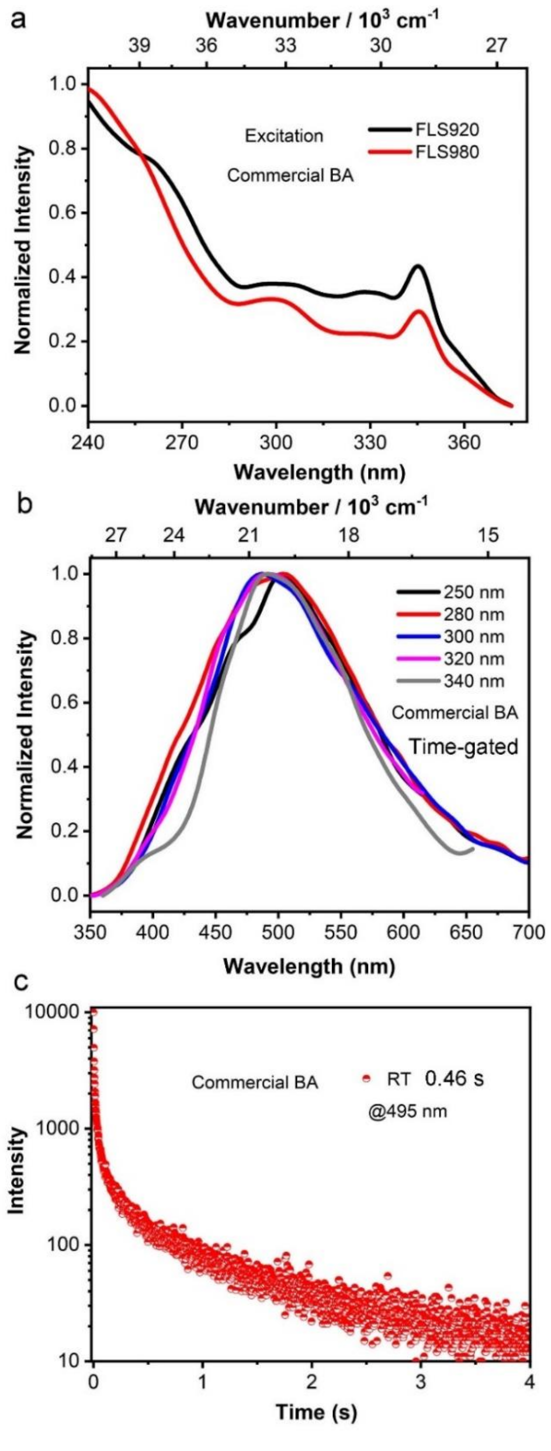

Figure S11. Normalized (a) excitation (390 nm) recorded on Edinburgh Instruments FLS920 and FLS980 spectrophotometers, (b) time-gated (delay time: 1 ms) phosphorescence at different excitation wavelengths (FLS920), and (c) phosphorescence decay (495 nm, λ_{ex} = 280 nm, FLS920) of commercial BA (Sigma-Aldrich: 99.999%) at room temperature. The dependence of the PL spectra on the excitation wavelength points to heterogeneity of the sensitizer, either from different local environments in the sample or, possibly, different contaminants. This might also vary somewhat between batches.

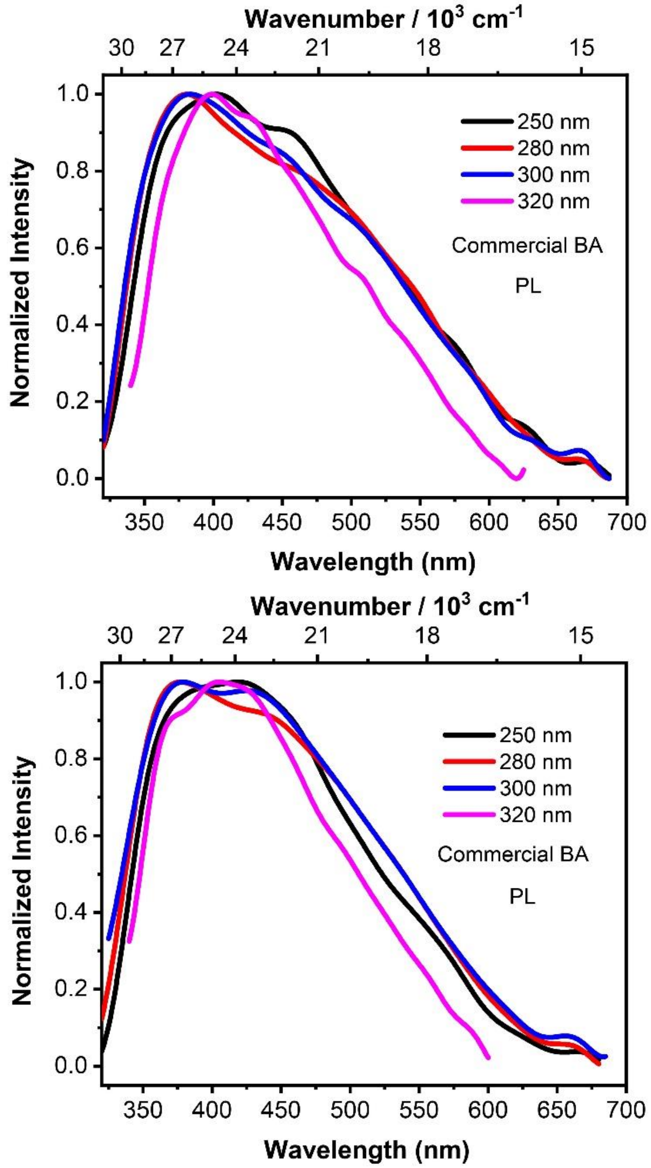

Figure S12. Normalized photoluminescence spectra of commercial BA (Sigma-Aldrich: 99.999%) at different excitation wavelengths at room temperature on two different spectrophotometers (top: Edinburgh Instruments FLS920; bottom Edinburgh Instruments FLS980).

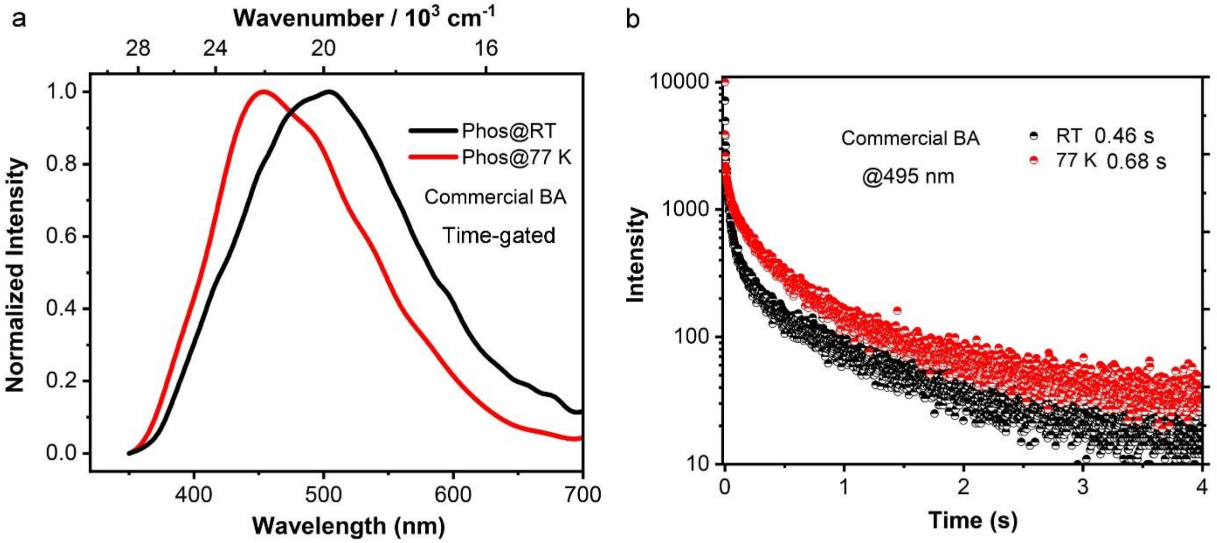

Figure S13. (a) Normalized time-gated (delay time: 1 ms) phosphorescence spectra of commercial BA (Sigma-Aldrich: 99.999%) at room temperature and 77 K, (b) Decays of phosphorescence (495 nm) at room temperature and 77 K (λ_{ex} = 280 nm). Recorded on the FLS920 spectrophotometer.

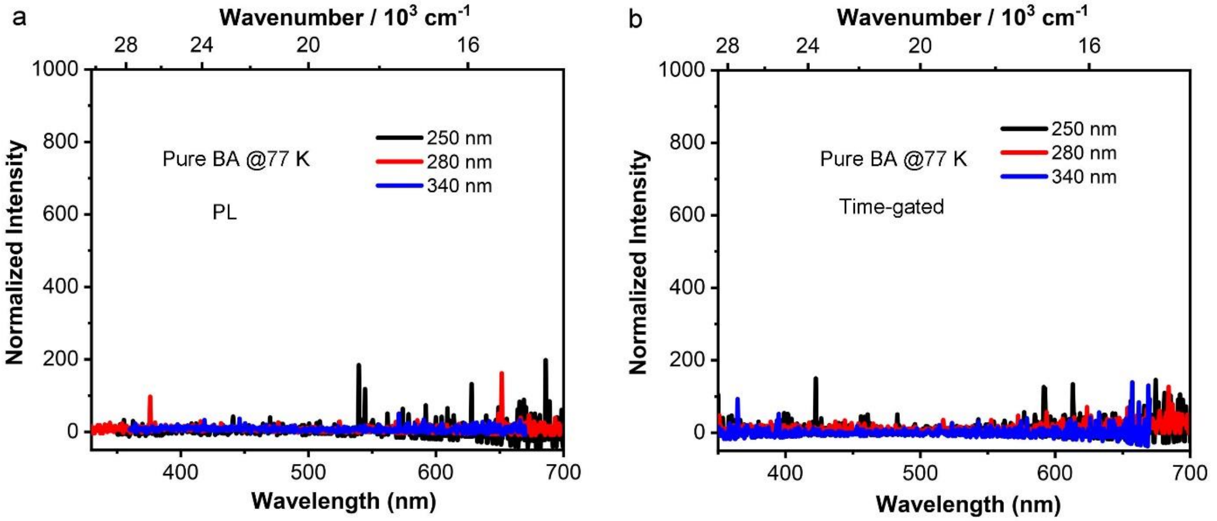

Figure S14. Photoluminescence (a) and time-gated (delay time = 1 ms) emission (b) spectra of pure BA in the crystalline state at different excitation wavelengths at 77 K. Recorded on the FLS920 spectrophotometer.

IV. Results of the quantum chemical computations

Table S1. Lowest excited singlet and triplet states (S_1, T_1) as calculated by TD-DFT, presenting vertical transition energies E_{vert} (and wavelength λ_{vert}), oscillator strength and composition (H = HOMO, L = LUMO).

	State	E_{vert} / eV	λ_{vert} / nm	Osc. Streng.	Main CI contribution
1x1	S_1	8.14	152	0.0000	H→L (88%)
	T_1	7.77	160	-	H→L (76%)
1x2	S_1	7.96	156	0.0000	H→L (90%)
	T_1	7.75	160	-	H→L (79%)
2x2	S_1	7.59	163	0.0006	H→L (91%)
	T_1	7.46	166	-	H→L (71%)
4x2	S_1	7.26	171	0.0004	H→L (91%)
	T_1	7.17	173	-	H→L (85%)
8x2	S_1	7.21	172	0.0013	H→L (80%)
	T_1	7.13	174	-	H→L (73%)
2x1	S_1	7.75	160	0.0008	H→L (94%)
	T_1	7.50	165	-	H→L (86%)
4x1	S_1	7.52	165	0.0004	H→L (82%)
	T_1	7.32	169	-	H→L (75%)
8x1	S_1	7.46	166	0.0005	H→L (69%)
	T_1	7.28	170	-	H→L (59%)
16x1	S_1	7.38	168	0.0013	H→L (45%)
					H→L+2 (28%)
	T_1	7.19	173	-	H→L (36%)
					H→L+2 (26%)

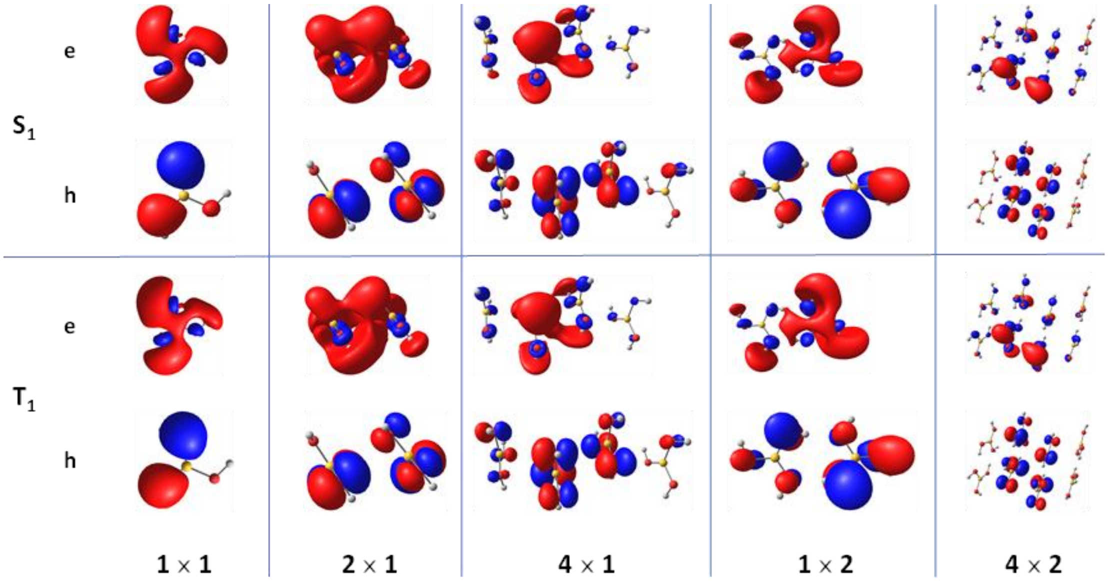

Figure S15. DFT calculated natural transition orbitals (NTOs) for the S_1 and T_1 states of $B(OH)_3$ and its clusters, representing holes (h) and electrons (e).

Excited State Relaxation. In Figure S19 of the Supporting Information of Ref. [18], the authors present calculations of the relaxed excited states for BA_x (x = 1,2,6) in the gas phase, being about 6 eV lower than the vertical absorption; this was, however, not further discussed by the authors. We thus repeated the calculations, optimizing the S_1 state of BA in the gas phase at the TD-DFT level; this indeed gives an adiabatic energy of only $E_{ad}(S_0 \leftrightarrow S_1) = 2.39$ eV, compared with a vertical absorption of $E_{vert}(S_0 \rightarrow S_1) = 8.14$ eV. However, closer inspection of the optimized S_1 geometry (see Figure S16) shows a considerable, partial heterolytic dissociation of one O···H bond as supported by the Mulliken charges analysis in Figure S16; a non-bonding distance of 1.747 Å was obtained (compared with 0.962 Å in S_0), while the respective B–O bond length is not significantly shortened. This photo*chemical* reaction is the reason for the very low energy of S_1, unnoticed in Ref. [18]. The same result was obtained by implicit solvent inclusion via PCM. Furthermore, in order to exclude a methodological failure, we performed CASSCF (complete active space self-consistent field) calculations, which confirmed the partial dissociation in S_1. We note that deprotonated BA species were indeed experimentally observed in the gas phase (by negative ion mass spectrometry),[19] as well as in crystalline Brønsted base conjugates.[20] Therefore, in a further step, we investigated whether such photochemical reaction is a possible pathway also in solid state BA; this, however, requires the explicit inclusion of the crystal environment. For this, we performed QM/MM (quantum mechanics/molecular mechanics) calculations of crystalline (and amorphous) BA (Figure S17), which, in fact, also predicts the partial dissociation of BA in the S_1 state. We emphasize, however, that this (potential) photochemical path can only be activated by irradiation of the sample in the far UV (> 7 eV), and not in the 250-370 nm range;

in fact, in the near/middle UV, pure BA does not absorb and, consequently, does not emit, as unambiguously demonstrated by the combined experimental and computational work of the present paper.

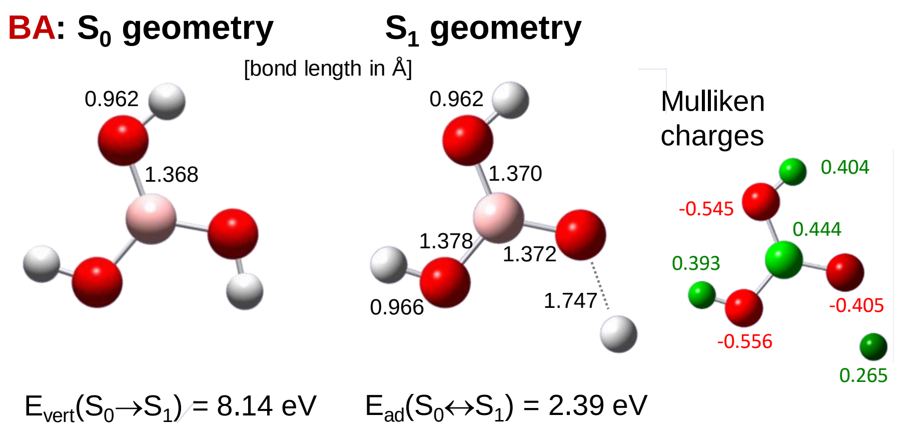

Figure S16. (TD)DFT-optimized geometries of BA in the gas phase for the ground state (S_0) and first excited singlet state (S_1) with bond lengths (in Å); Mulliken charges in S_1, vertical absorption energy E_{vert} and adiabatic energy E_{ad}.

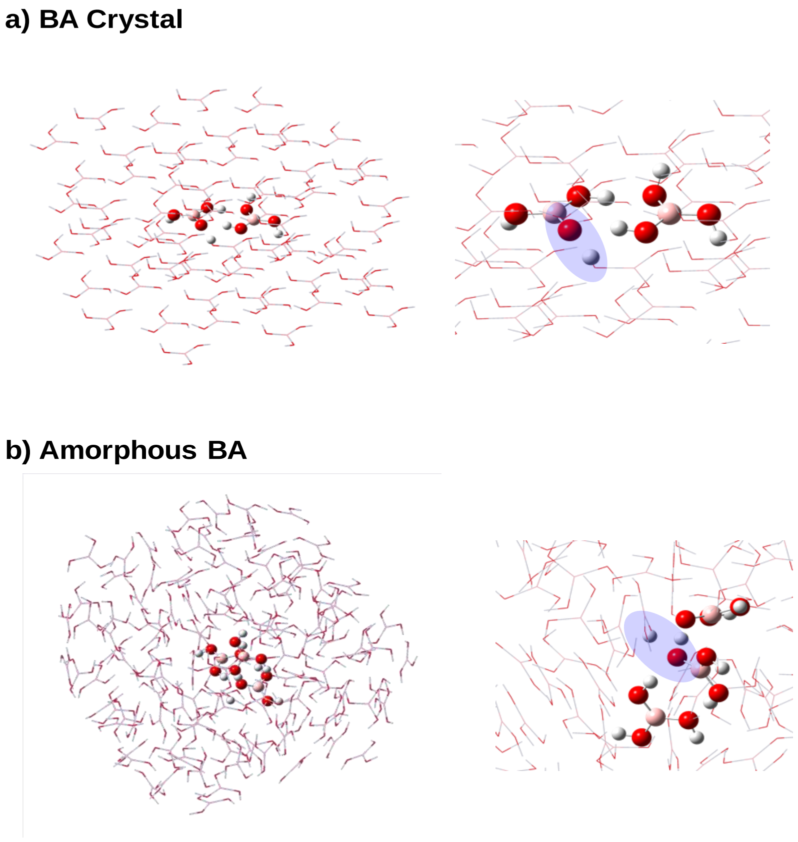

Figure S17. Optimized S_1 geometries of BA by QM/MM calculations; a) BA crystal, b) amorphous BA; partially dissociated bonds are highlighted in the zoom-in on the right.

V. Single-crystal X-ray diffraction

Table S2. Single-crystal X-ray diffraction data and refinement details of $B(OH)_3$ at 100 K and 296 K in comparison with literature data at 105 K[6].

Data	$B(OH)_3$	$B(OH)_3$	$B(OH)_3$ (reported)[6]
CCDC number	2122545	2122546	ICSD number: 61354
Empirical formula	BH_3O_3	BH_3O_3	BH_3O_3
Formula weight / g·mol^{-1}	61.83	61.83	61.83
T / K	100(2)	296(2)	105
Radiation, λ / Å	Cu-K_{α}, 1.54184	Mo-K_{α}, 0.71073	Mo-K_{α}, 0.71073
Crystal size / mm³	0.021×0.104×0.202	0.416×0.309×0.302	0.15×0.10×0.08
Crystal color, habit	colourless plate	colourless block	-[a]
μ / mm^{-1}	1.453	0.155	0.151
Crystal system	triclinic	triclinic	triclinic
Space group	$P\bar{1}$	$P\bar{1}$	$P\bar{1}$
a / Å	7.0181(3)	7.032(2)	7.0187 (14)
b / Å	7.0360(4)	7.045(4)	7.035 (2)
c / Å	6.3469(5)	6.5737(19)	6.5472 (12)
α / °	92.481(5)	92.51(3)	92.49 (12)
β / °	101.423(5)	101.19(2)	101.46 (2)
γ / °	119.759(5)	119.814(17)	119.76 (2)
Volume / Å^3	262.97(3)	273.4(2)	271.19 (1)
Z	4	4	4
ρ_{calc} / g·cm^{-3}	1.562	1.502	1.562
F(000)	128	128	128
θ range / °	7.219 – 74.402	3.203 – 26.368	up to 55.53
Reflections collected	5615	4287	15761
Unique reflections	1063	1100	1673 [I>2σ(I)] $34.65 < \theta < 55.53$
Parameters / restraints	79 / 0	79 / 0	73 / 0
GooF on F^2	1.067	1.247	1.004
R_1 [I>2σ(I)]	0.0426	0.0471	0.042
wR_2 (all data)	0.1252	0.1577	0.040 (wR)
Max. / min. residual electron density / e·Å^{-3}	0.276 / –0.395	0.224 / –0.223	0.70 / -0.70

[a] The corresponding information is not provided in the published reference.[6]

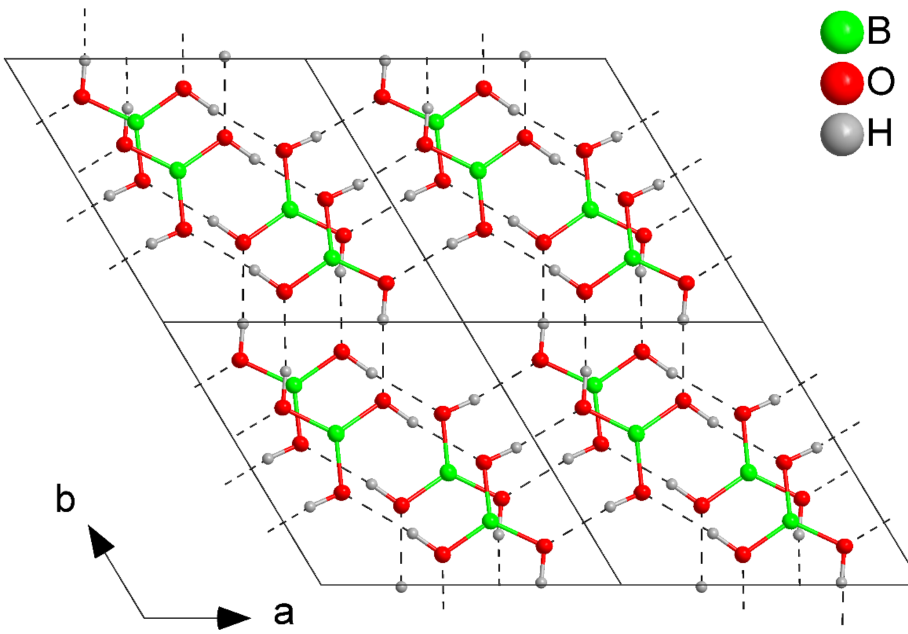

Figure S18. Eight unit cells (2×2×2) of the crystal structure of boric acid showing the hydrogen-bonding network within the sheets at 100 K.

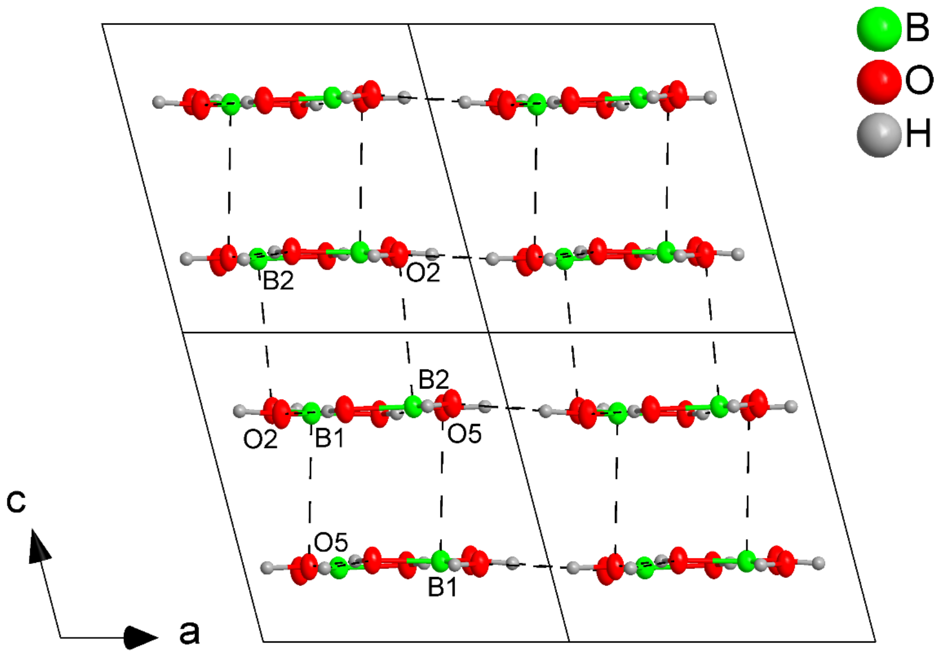

Figure S19. Four unit cells (2×1×2) of the crystal structure of boric acid projected along the *b* axis at 100 K. The closest interplanar B–O contacts (Å) are illustrated by dashed lines: B1–O5 = 3.064(2), B2–O2 = 3.031(2).

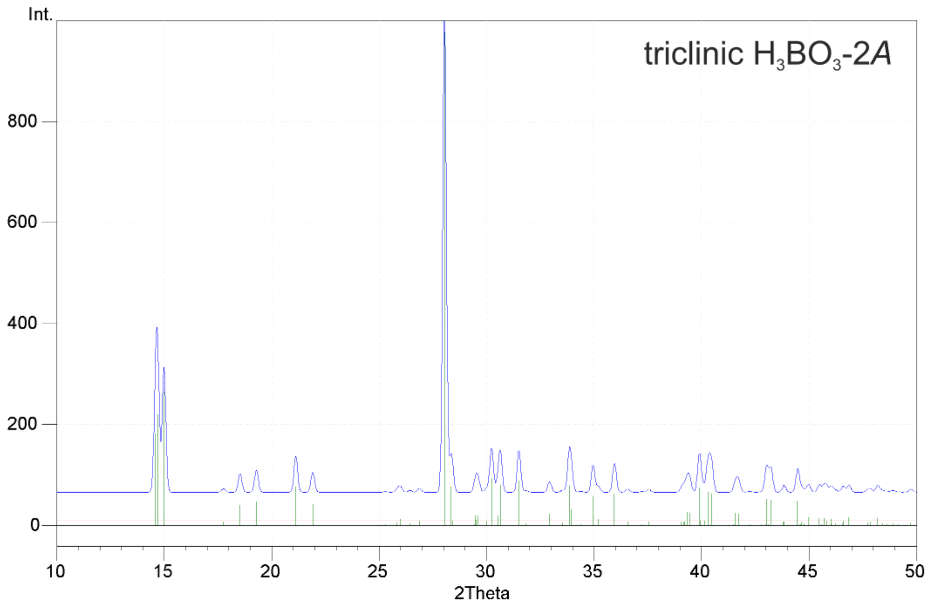

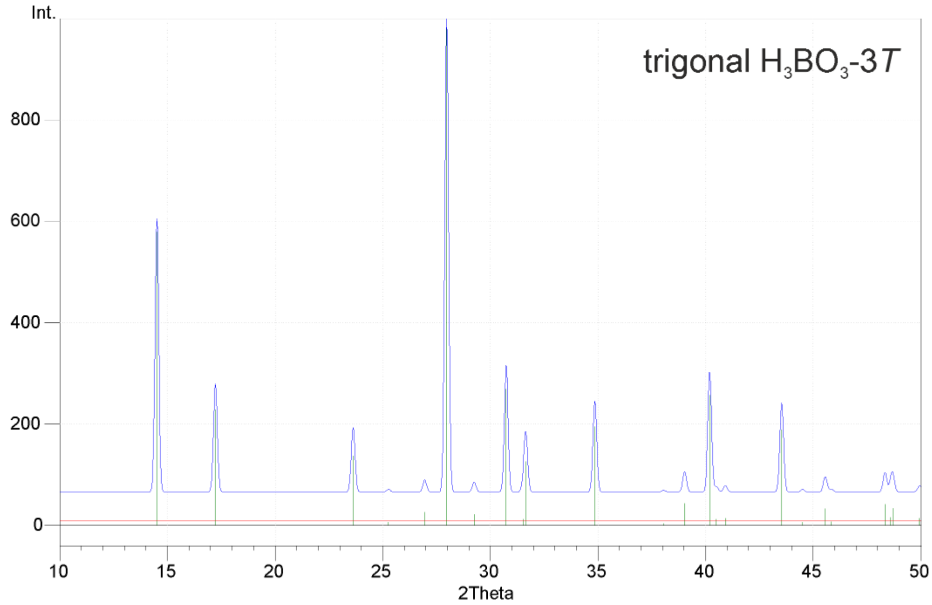

Figure S20. Simulated powder X-ray diffraction patterns (blue lines) and reflection positions (green lines) of the room temperature structures of triclinic boric acid, H_3BO_3-2*A* (top, our study) and trigonal boric acid, H_3BO_3-3*T* (bottom, ICSD-281322). Simulations were performed for Cu-Kα_1 radiation with a wavelength λ = 1.5406 Å using the Diamond program.[7]

VI. ^{11}B NMR spectra

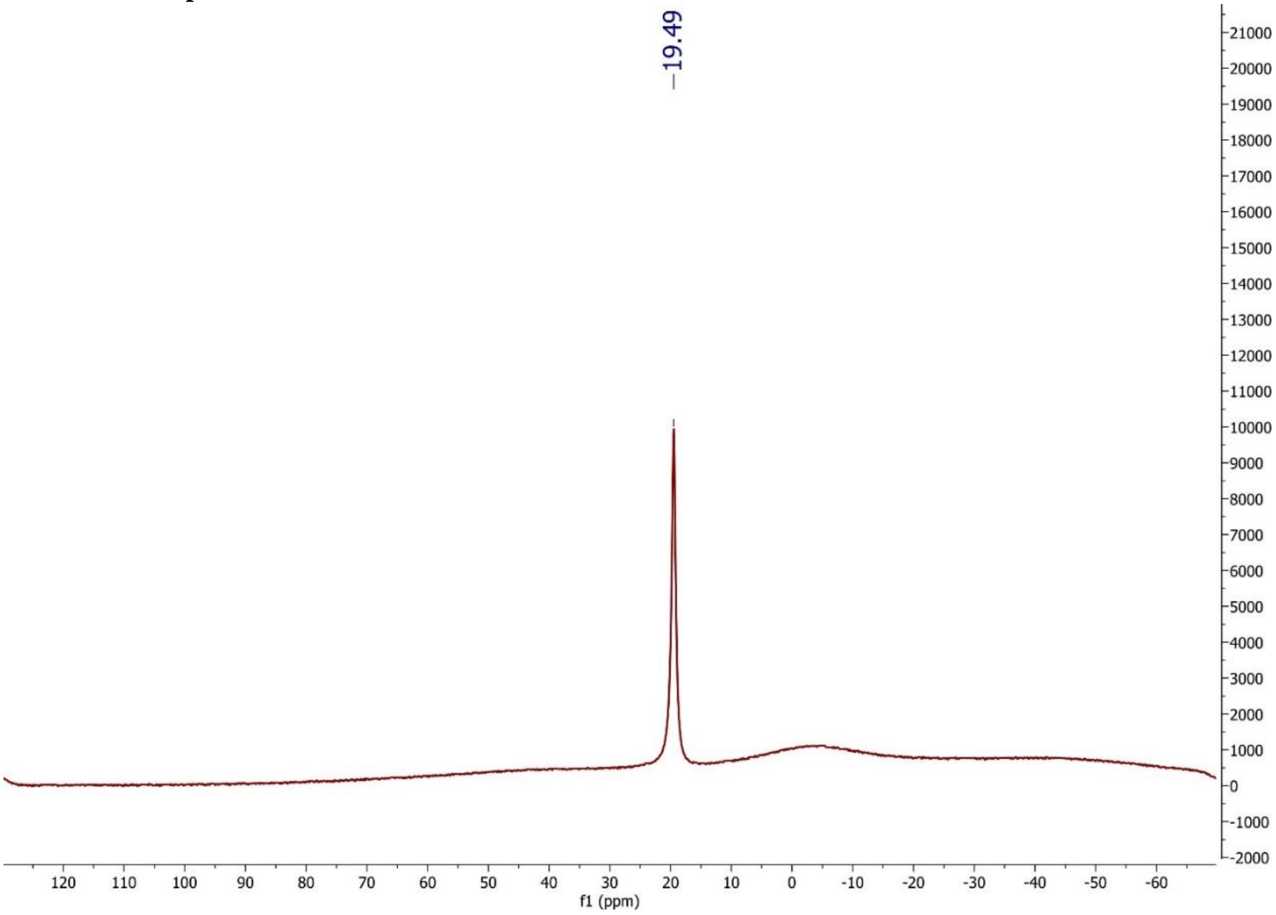

Figure S21. ^{11}B NMR spectrum of synthesized $B(OH)_3$ in D_2O (96 MHz).

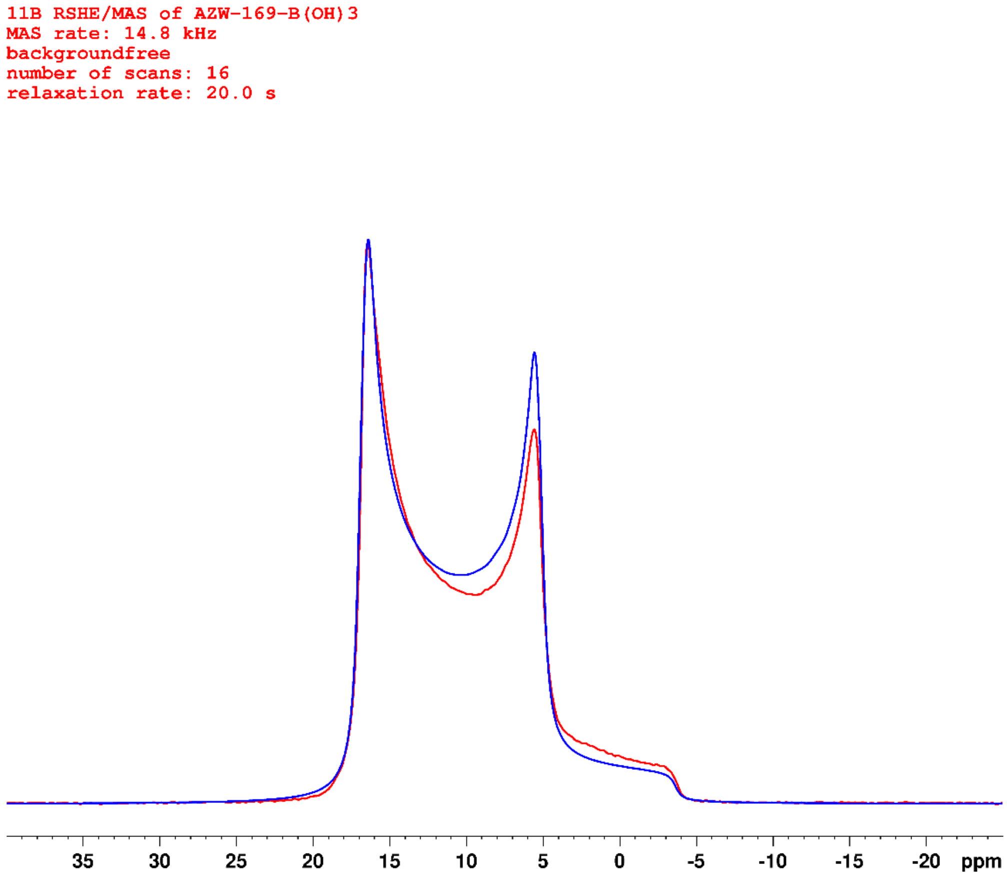

Figure S22. ^{11}B solid state NMR spectrum (blue = fit; red = measured spectrum) of synthesized $B(OH)_3$ (MAS: 14.8 KHz).

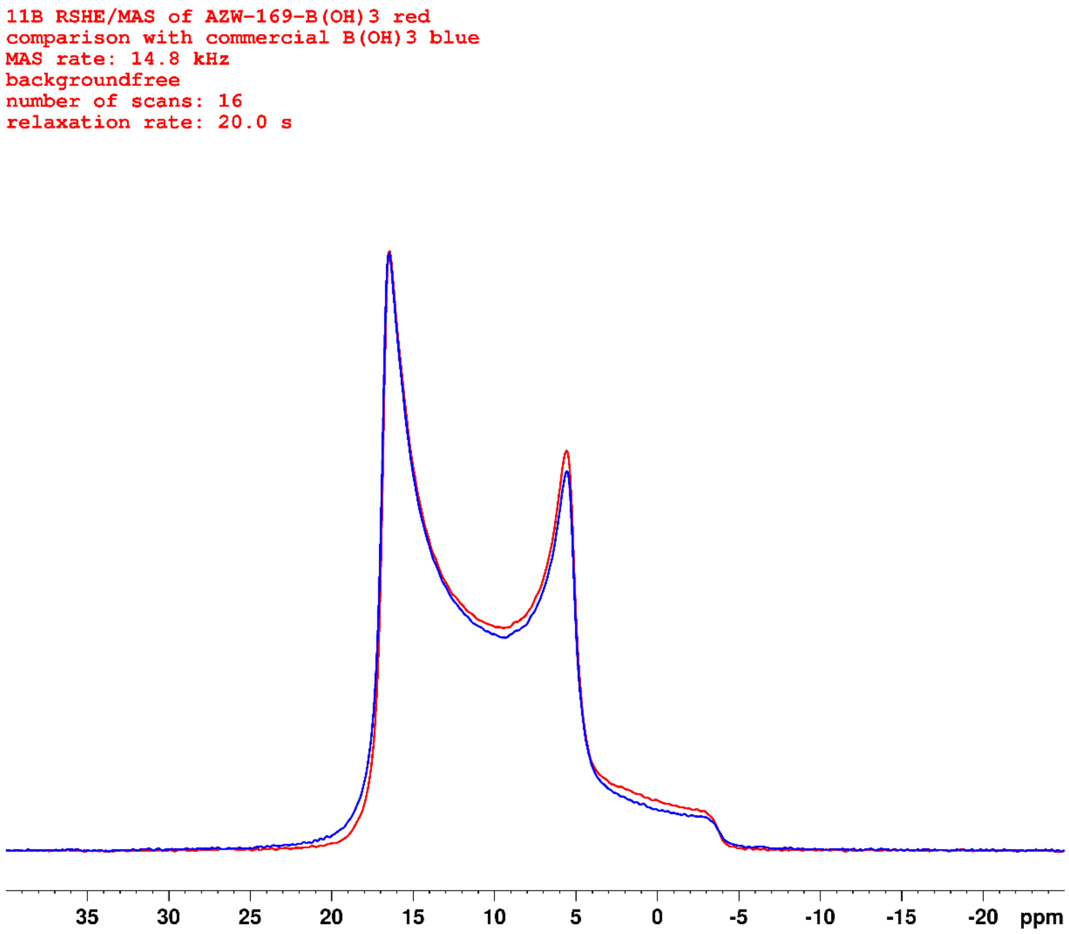

Figure S23. ^{11}B solid state NMR spectra of $B(OH)_3$ (blue = commercial; red = synthesized) (MAS: 14.8 KHz).

VII. References

[1] J. Rohonczy, SOLA – Solid Lineshape Analysis Version 2.2.4, Bruker Biospin, Rheinstetten, Germany 2013.

[2] J. Gierschner, J. Shi, B. Milián-Medina, D. Roca-Sanjuán, S. Varghese, S.-Y. Park, *Adv. Opt. Mater*. **2021**, *9*, 20002251.

[3] G. M. Sheldrick, *Acta Crystallogr. A Found Adv.* **2015**, *71*, 3-8.

[4] G. M. Sheldrick, *Acta Crystallogr. A* **2008**, *64*, 112-122.

[5] C. B. Hübschle, G. M. Sheldrick, B. Dittrich, *J. Appl. Crystallogr.* **2011**, *44*, 1281-1284.

[6] M. Gajhede, S. Larsen, S. Rettrup, *Acta Crystallogr.* **1986**, *B42*, 545–552.

[7] K. D. Brandenburg, Diamond (version 4.6.6), Crystal and Molecular Structure Visualization, Crystal Impact, H. Putz & K. Brandenburg GbR, Bonn (Germany), 2017.

[8] C. F. Macrae, I. J. Bruno, J. A. Chisholm, P. R. Edgington, P. McCabe, E. Pidcock, L. Rodriguez-Monge, R. Taylor, J. van de Streek, P. A. Wood, *J. Appl. Crystallogr.* **2008**, *41*, 466-470.

[9] O. V. Dolomanov, L. J. Bourhis, R. J. Gildea, J. A. K. Howard, H. Puschmann, *J. Appl. Crystallogr.* **2009**, *42*, 339-341.

[10] S. Dapprich, I. Komáromi, K. S. Byun, K. Morokuma, M. J. Frisch, A new ONIOM Implementation in Gaussian98. Part I. The Calculation of Energies, Gradients, Vibrational Frequencies and Electric Field Derivatives. *J. Mol. Struct.: THEOCHEM* **1999**, *461-462*, 1-21.

[11] M. Wykes, R. Parambil Mangattu, D. Beljonne, J. Gierschner, *J. Chem. Phys.* **2015**, *143*, 114116.

[12] M. Wykes, S. K. Park, S. Bhattacharyya, S. Varghese, J. E. Kwon, D. R. Whang, I. Cho, R. Wannemacher, L. Lüer, S. Y. Park, J. Gierschner, *J. Phys. Chem. Lett.* **2015**, *6*, 3682–3687.

[13] Gaussian 09, Revision D.01, M. J. Frisch, G. W. Trucks, H. B. Schlegel, G. E. Scuseria, M. A. Robb, J. R. Cheeseman, G. Scalmani, V. Barone, B. Mennucci, G. A. Petersson, H. Nakatsuji, M. Caricato, X. Li, H. P. Hratchian, A. F. Izmaylov, J. Bloino, G. Zheng, J. L. Sonnenberg, M. Hada, M. Ehara, K. Toyota, R. Fukuda, J. Hasegawa, M. Ishida, T. Nakajima, Y. Honda, O. Kitao, H. Nakai, T. Vreven, J. A. Montgomery, Jr., J. E. Peralta, F. Ogliaro, M. Bearpark, J. J. Heyd, E. Brothers, K. N. Kudin, V. N. Staroverov, T. Keith, R. Kobayashi, J. Normand, K. Raghavachari, A. Rendell, J. C. Burant, S. S. Iyengar, J. Tomasi, M. Cossi, N. Rega, J. M. Millam, M. Klene, J. E. Knox, J. B. Cross, V. Bakken, C. Adamo, J. Jaramillo, R. Gomperts, R. E. Stratmann, O. Yazyev, A. J. Austin, R. Cammi, C. Pomelli, J. W. Ochterski, R. L. Martin, K. Morokuma, V. G. Zakrzewski, G. A. Voth, P. Salvador, J. J. Dannenberg, S. Dapprich, A. D. Daniels, O. Farkas, J. B. Foresman, J. V. Ortiz, J. Cioslowski, and D. J. Fox, Gaussian, Inc., Wallingford CT, **2013**.

[14] I. Fdez. Galván, M. Vacher, A. Alavi, C. Angeli, F. Aquilante, J. Autschbach, J. J. Bao, S. I. Bokarev, N. A. Bogdanov, R. K. Carlson, L. F. Chibotaru, J. Creutzberg, N. Dattani, M. G. Delcey, S. S. Dong, A. Dreuw, L. Freitag, L. M. Frutos, L. Gagliardi, F. Gendron, A. Giussani, L. González, G. Grell, M. Guo, C. E. Hoyer, M. Johansson, S. Keller, S. Knecht, G. Kovačević, E. Källman, G. Li Manni, M. Lundberg, Y. Ma, S. Mai, J. P. Malhado, P. Å. Malmqvist, P. Marquetand, S. A. Mewes, J. Norell, M. Olivucci, M. Oppel, Q. M. Phung, K. Pierloot, F. Plasser, M. Reiher, A. M. Sand, I. Schapiro, P. Sharma, C. J. Stein, L. K. Sørensen, D. G. Truhlar, M. Ugandi, L. Ungur, A. Valentini, S. Vancoillie, V. Veryazov, O. Weser, T. A. Wesołowski, P. O. Widmark, S. Wouters, A. Zech, J. P. Zobel, and R. Lindh, *J. Chem. Theory Comput.* **2019**, *15*, 5925.

[15] F. Aquilante, J. Autschbach, A. Baiardi, S. Battaglia, V. A. Borin, L. F. Chibotaru, I. Conti, L. De Vico, M. Delcey, I. F. Galván, N. Ferré, L. Freitag, M. Garavelli, X. Gong, S. Knecht, E. D. Larsson, R. Lindh, M. Lundberg, P. Å. Malmqvist, A. Nenov, J. Norell, M. Odelius, M. Olivucci, T. B. Pedersen, L. Pedraza-González, Q. M. Phung, K. Pierloot, M. Reiher, I. Schapiro, J. Segarra-Martí, F. Segatta, L. Seijo, S. Sen, D. C. Sergentu, C. J. Stein, L. Ungur, M. Vacher, A. Valentini, and V. Veryazov, *J. Chem. Phys.* **2020**, *152*, 214117.

[16] P. Å. Malmqvist and B. O. Roos, *Chem. Phys. Lett.* **1989**, *155*, 189.

[17] J. P. Zobel, P. O. Widmark, and V. Veryazov, *J. Chem. Theory Comput.* **2020**, *16*, 278.

[18] H. Zheng, P. Cao, Y. Wang, X. Lu, P. Wu, *Angew. Chem. Int. Ed.* **2021**, *60*, 9500-9506; *Angew. Chem.* **2021**, *133*, 9586-9592.

[19] M. Attinà, F. Cacace, G. Occhiucci, A. Ricci, *Inorg. Chem.* **1992**, *31*, 3114-3117.

[20] C. C. Freyhardt, M. Wiebcke, *J. Chem. Soc., Chem. Commun.*, **1994**, 1675-1676.

VIII. Certificate of Analysis for the commercial sample of BA

Sigma-Aldrich

3050 Spruce Street, Saint Louis, MO 63103, USA
Website: www.sigma-aldrich.com
Email USA: techserv@sial.com
Outside USA: eurtechserv@sial.com

Certificate of Analysis

**Product Name :**	**Boric acid 99.999% trace metals basis**
**Product Number :**	**202878-VAR**
**Batch Number :**	**0000107066**
Source Batch :	0000104141
CAS Number :	10043-35-3
Molecular Formula :	H_3BO_3
Formula Weight :	61.83
Quality Release Date :	30 Sep 2020

Test	Specification	Result
Appearance (Color)	White	White
Appearance (Form)	Crystalline Powder	Crystalline Powder
Infrared Spectrum	Conforms to Structure	Conforms
Titration with NaOH	≥ 98.0 %	100.4 %
ICP Major Analysis	Confirmed	Confirmed
Confirms Boron Component		
Purity	Meets Requirements	Meets Requirements
99.999% Based on Trace Metals Analysis		
Trace Metal Analysis	≤ 15.0 ppm	8.1 ppm
Aluminum (Al)		7.0 ppm
Iron (Fe)		0.2 ppm
Potassium (K)		0.8 ppm
Sodium (Na)		0.1 ppm

Niraj K. Singh

Niraj K. Singh, Manager
Site
Urbana, Illinois
US

Sigma-Aldrich warrants, that at the time of the quality release or subsequent retest date this product conformed to the information contained in this publication. The current Specification sheet may be available at Sigma-Aldrich.com. For further inquiries, please contact Technical Service. Purchase must determine the suitability of the product for its particular use. See reverse side of website or packing slip for additional terms and conditions of sale

Version Number: 01 Doc: 1051144

The branding on the header and/or footer of this document may temporarily not visually match the product purchased as we transition our branding. However, all of the information in the document regarding the product remains unchanged and matches the product ordered.
For further information please contact mlsbranding@sial.com
